# Investigating environmental effects on phonology using diachronic models

**DOI:** 10.1017/ehs.2023.33

**Published:** 2024-01-03

**Authors:** Frederik Hartmann, Seán G. Roberts, Paul Valdes, Rebecca Grollemund

**Affiliations:** 1 University of North Texas, Denton, Texas, USA; 2 Cardiff University, Cardiff, UK; 3 University of Bristol, Bristol, UK; 4 University of Missouri-Columbia, Columbia, Missouri, USA

**Keywords:** Phylogenetics, climate, tones, humidity

## Abstract

Previous work has proposed various mechanisms by which the environment may affect the emergence of linguistic features. For example, dry air may cause careful control of pitch to be more effortful, and so affect the emergence of linguistic distinctions that rely on pitch such as lexical tone or vowel inventories. Criticisms of these proposals point out that there are both historical and geographic confounds that need to be controlled for. We take a causal inference approach to this problem to design the most detailed test of the theory to date. We analyse languages from the Bantu language family, using a prior geographic–phylogenetic tree of relationships to establish where and when languages were spoken. This is combined with estimates of humidity for those times and places, taken from historical climate models. We then estimate the strength of causal relationships in a causal path model, controlling for various influences of inheritance and borrowing. We find no evidence to support the previous claims that humidity affects the emergence of lexical tone. This study shows how using causal inference approaches lets us test complex causal claims about the cultural evolution of language.

## Graphical abstract

**Figure:**
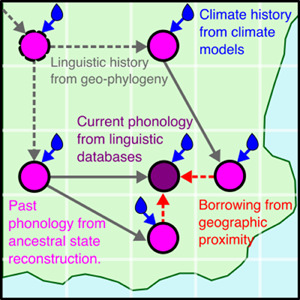

**Social media summary:** A test of whether speech sounds adapt to humidity using causal methods to reconstruct cultural and environmental history

## 1. Introduction

One of the major challenges for linguistics is not just to describe the variation between languages, but to explain it. While there may be no strong, deterministic causal effects in cultural systems, statistical patterns demand explanations (Bickel & Hickey, 2017). For example, one pattern that has captured researchers’ attention recently is co-variation between linguistic features and environmental features (Bentz, Dediu, Verkerk, & Jäger, 2018). However, linguists are a long way from demonstrating solid evidence for causal effects between these domains since the relationships between them are very complex. In this paper, we present a causal inference approach to this kind of relationship, making two novel contributions. First, we show that it is possible to match the estimates of temporal and spatial locations of historical languages to historical estimates of environmental variables. Secondly, we use methods from causal inference to design a novel regression analysis that can control for various confounding effects such as inheritance, borrowing and geographic proximity. We focus on claims that, over thousands of years, properties of the environment can affect the sound structures of a spoken language (Everett, 2021), specifically the effect of humidity on lexical tone (Everett, Blasi, & Roberts, 2015) and vowel inventories (Everett, 2017). Our aim is to contribute to the methodology for testing whether these claims are robust or based on spurious correlations.

Hypotheses about the effects of the climate on speech sounds have been around since the 1780s (de Rivarol, 1784; Pinkerton, 1789). However, as with many older studies of cross-linguistic differences, the motivations of these studies should be questioned in the light of the racist and colonialist attitudes of the time (see Winter & Wedel, 2016). In contrast, the modern approach to the idea can be traced to a very standard linguistic explanation: the principle of least effort. This states that speech sounds and other language structures will adapt to be understandable with the minimum production effort (Bybee, 2006; Zipf, 1949). Previous studies focused on the relationship between temperature and the number of meaningful vowel distinctions in a language (Ember & Ember, 2007; Fought, Munroe, Fought, & Good, 2004), with more recent studies controlling for various historical confounds and using more general measures of the acoustic sonority of a language's sound inventory (Maddieson, 2018; Maddieson & Coupé, 2015).

Caleb Everett extended this line of research using evidence from anatomical responses of the speech organs to the climate in order to bridge the theoretical gap around production effort (Everett, 2013, 2021). He argued for the following causal chain: ambient humidity → vocal fold desiccation → voicing control → linguistic dependence on voicing. First, inhaling dry air desiccates the vocal folds (the organ that creates speech sounds by vibration which contribute to vowels, voiced consonants and pitch). This makes precise control of voicing more effortful. The principle of least effort would then predict that the languages in dry environments would evolve over thousands of years to rely less on voicing as a distinctive characteristic.

Everett et al. (2015) tested a prediction of this causal chain based on lexical tone. All languages make use of pitch to convey meaning, for example in English rising pitch at the end of a sentence can mark the pragmatic distinction between a question and a statement. However, some languages such as Mandarin or Cantonese make use of lexical tone, where distinctions in pitch are used distinguish lexical meanings (e.g. the same phonemes with rising or falling pitch can be semantically unrelated words). Languages can vary from having two distinctions in tone up to complex systems that use combinations of pitch contours to create 12 distinctions (Donohue, Hetherington, McElvenny, & Dawson., 2013). Lexical tone is used in roughly a third of the world's languages, and is common in sub-Saharan Africa, Asia, the Pacific and the Americas, and is also used in some European languages such as Norwegian (Maddieson, 2013; Moran & McCloy, 2019).

Everett et al. argued that, everything else being equal, successful communication in a language with lexical tone depends more on precise control of pitch to distinguish lexical meanings than a language that does not use lexical tone. Because dry air makes careful control of pitch more effortful, lexical tone would be less likely to emerge or survive in dry locations. Therefore, the prediction is that languages with lexical tone will be relatively less common in dry regions of the world. Everett et al. (2015) found supporting correlational evidence for this in a global sample of languages. An extension by Everett (2017) also tested the same prediction for the general dependence on vowels compared with consonants measured by the ‘vowel index’ (the proportion of vowels to consonants in words from basic vocabulary lists).

The response to this work was very critical. Several commentaries argued that any effect of the ambient humidity would be undetectable owing to compensating mechanisms in the vocal tract (De Boer, 2016; Ladd, 2016), or material culture (Donohue, 2016) or adjustments to perception during processing (Mendívil-Giró, 2018). More critically still, subsequent studies failed to replicate the findings using alternative analyses (Hammarström, 2016) or data sources (Roberts, 2018). Furthermore, while the original study attempted to control for historical relationships between data points, there were several confounds that were not accounted for, as we discuss further below.

More recent work includes improvements in the measurement of climatic and linguistic variables. Maddieson and Benedict (2023) measure the correlations between various environmental and linguistic variables. The methods include advanced geographic methods for defining the geographic ranges of languages and integrating the temporal variability of environmental variables. Significant raw correlations are replicated between humidity and vowel index, and between humidity and number of tones. However, controls for historical and geospatial autocorrelation were not applied. Liang et al. (2023) analyse the relationships between humidity, voice quality and number of tones in 997 language varieties in China from over a million voice recordings. The results show that lower humidity is associated with poorer voice quality (more audio jitter and shimmer), and that poorer voice quality is associated with the variety having fewer contrastive tones. However, this study did not attempt to model changes over time, nor did it take into account detailed historical and geographic relations between languages.

Despite the range of problems faced by this line of research, or perhaps because of them, the studies on tone and climate and similar studies using cross-cultural comparison (see above and Atkinson, 2011; Dediu and Ladd, 2007; Lupyan and Dale, 2010; Regier, Carstensen, and Kemp, 2016) seemed to act as catalysts for change. They encouraged debate about the causal processes of language change and how to estimate causal effects between language structures and language-external forces from cross-cultural data. That debate has focused on three main challenges for empirical work in this area, which we explain below, and which we aim to tackle in this paper.

### 1.1. Challenges for inferring environmental effects on language

The first challenge relates to data. We want to estimate changes in the past from data collected in the present. Current data only provide a current snapshot of languages, whereas the ideal evidence would be how languages change over time. Phylogenetic methods for estimating ancestral states of cultural features have helped reconstruct past states (Jordan, Gray, Greenhill, & Mace, 2009; Mace & Holden, 2005; Moran, Grossman, & Verkerk, 2021; Phillips & Bowern, 2022). Moran (2016) discusses specific problems in relation to estimating the effect of humidity on tone, including the complexity of the historical process of gaining and losing lexical tone. Moran questions the reliability of the original data sources. Since then, resources like the PHOIBLE database have provided an open-source repository for data on phonological inventories (Moran & McCloy, 2019). Furthermore, the issue also exists for language-external variables such as humidity: the humidity of an area may change over the time scale that is being considered, and indeed the speakers of a language may move to areas with different climatic conditions.

The second challenge is controlling for historical relatedness. Languages derived from the same ancestor will tend to inherit similar properties, and so represent non-independent observations. Without controlling for this, estimates of correlations involving cultural traits can be inflated (phylogenetic auto-correlation; see Roberts & Winters, 2013). Bayesian phylogenetic trees of language families provide the necessary information to control for this effect. However, few language families have robust, dated phylogenies, so many studies using global language rely on mixed effects models, controlling only for which language family a language belongs to.

The third challenge is controlling for geographic proximity, which has two issues. The first is the case of borrowing between languages in contact. Moran, Lester, and Grossman (2021) estimate the effect of borrowing on speech sounds to be considerable, and M.-h. Zhang, Pan, Yan, and Jin (2018) use admixture models to show that phonemic features, including tone, are likely to have undergone borrowing in the evolution of Chinese dialects. Therefore, borrowing represents a major confound for the link between humidity and tone (Collins, 2016, 2017). The second, related issue of this challenge is that languages in similar locations will tend to have similar physical environments (geographic auto-correlation; Bromham, Hua, Cardillo, Schneemann, and Greenhill, 2018). Hartmann (2022) shows that controlling for spatial proximity removes the apparent effect of humidity on lexical tone.

The three challenges are well known in linguistics, but methods for solving them all at the same time are still emerging (see e.g. Guzmán Naranjo & Mertner, 2022; Murawaki & Yamauchi, 2018). In this paper, we address each of these challenges using insights from causal inference.

### 1.2. Causal inference and cultural evolution

Before explaining our approach, we briefly cover causal inference and how it applies to the study of linguistic typology. Causal inference is an approach to estimating the strength of causal effects (Li, Ding, & Mealli, 2023; Pearl et al., 2000). Given various assumptions and an explicit model of causal relations between key variables, it is possible to calculate the strength of causal effects from observational data. One critical tool for causal inference is causal graphs (or ‘causal networks’ or ‘Bayesian networks’; Pearl, 1985) – visual representations of hypotheses about the causal relations between variables. Nodes represent variables and an arrow is drawn between two nodes to indicate the hypothesis that if the source variable were to change then the destination variable would also change. Causal graphs are underwritten by powerful mathematical axioms (Pearl, 1995) that can help identify confounding variables and design statistical tests and experiments (Pearl, Glymour, & Jewell, 2016). They are also effective ways of expressing and communicating hypotheses (Pearl & Mackenzie, 2018), and they are used in many fields including evolutionary linguistics (Roberts et al., 2020) and ecology (Sugihara et al., 2012).

A standard application of causal inference in social science is the potential outcomes model (Morgan & Winship, 2015). For linguistic cases, this approach would ask whether a language had a potentially different configuration if it had evolved under different conditions. We cannot observe this counterfactual language, but we can gather a large set of comparative data and estimate the average differences between languages in different conditions. That is, the relevant question for this paper is ‘If the speakers of language X had moved into a dry region instead of a humid region, how would the number of tones in the language change?’ Recently, the rise of large-scale, cross-cultural databases of the properties of language has made it possible to collect enough data to address this kind of question.

However, the causal inference approach was mainly designed for analysing cases in medical epidemiology. Various complications arise when looking at languages compared with a population of people, which require some discussion of concepts from various frameworks. For example, most epidemiological studies assume non-interference: the potential outcomes of one individual are independent of the treatment of another individual (Cox, 1958). This is not necessarily the case with languages, since linguistic features are inherited from ancestors and borrowed from neighbours (see below). Exchangeability is another important assumption (Saarela, Stephens, & Moodie, 2023), and it is not clear that one language is exchangeable for another. For example, a change in humidity alone may not be sufficient to cause a language to acquire tonal contrasts – tonogenesis (the process of a language gaining lexical tone) depends on particular linguistic conditions which may not be on a causal path from humidity (Michaud & Sands, 2020; Wu, Zhang, & Zhang, 2023). Furthermore, the effect of the environment on linguistic features may occur over long periods of time (see Section 4.2 for a note on consistency and temporal stability).

The solution is to take advantage of the reasonably specific historical relations that linguists have established between languages (compared with the common case in epidemiology where the structure of interference is unknown). This facilitates two strategies to overcome the issue of interference and exchangeability. First, one can assume that languages from different regions and different language families are independent, and consider the average effects across these groups. This is similar to how Hudgens and Halloran (2008) deal with interference in cases of infectious disease, although the question of exchangeability remains. The second strategy is to use the historical relations to estimate specific changes over time and test whether a change in one variable coincides with a change in another down the branches of a phylogenetic tree. The basis of this approach to causality is essentially Wiener–Granger causality (Granger, 1969; Wiener, 1956; see Turchin, 2018: 27; Ringen, Martin, & Jaeggi, 2021: 10) and addresses non-interference. More advanced uses of phylogenies may also address exchangeability. For example, Bromham, Hua, Fitzpatrick, and Greenhill (2015) analyse pairs of languages which have recently diverged from a common ancestor (thus being highly exchangeable because they were once the same). They found that the member of the pair that gained more speakers also gained more words than its sister. In some senses, this allows researchers to observe ‘conterfactual’ conditions: a sister language is its sibling as it could have been. A more general application of this approach is to see all languages in a language family that derive from a proto-ancestor as observable ‘potential’ outcomes under different conditions.

### 1.3. A causal approach to the challenges

This study takes a causal approach, including mapping the causal relationships of the original hypothesis using a causal graph, considering confounding factors, and dealing with relations in time. This allows us to design a statistical model which tackles each of the challenges above.

The data challenge is addressed by focusing on one language family where the historical and geographic relations have been estimated. We use a dated geo-phylo tree of Bantu languages (Grollemund et al., 2015). This estimates both the historical relationships between current languages, and the geographic locations of ancestors. Bantu is an ideal choice for the given research question, since it has variation in both humidity and tone (ensuring positivity, Hernan & Robins, 2020). Bantu covers a range of humidity conditions from rainforest to savannah (Bostoen et al., 2015) and these are spread throughout the phylogenetic tree. This might provide enough variation in climates to exhibit a pattern of replicated bursts of change across the phylogeny, an important factor in detecting evidence of correlated evolution (Maddison & FitzJohn, 2015). Additionally, its languages vary in the use of lexical tone and there is a linguistic reconstruction of tone in proto-Bantu. We also use a separate historical reconstruction of humidity from historical models of climate change from climatic science (Davies-Barnard, Ridgwell, Singarayer, & Valdes, 2017; Valdes et al., 2017). This allows us, for the first time that we are aware of, to combine historical estimates of both cultural and environmental variables. The use of the geo-phylo tree also lets us estimate and control for historical and geographic relationships, addressing these two challenges.

In the next section, we explain the reasoning behind the statistical model. In this way, we hope to make a methodological contribution, highlighting the importance of causal modelling in this field of research. Note that this study is based on claims and findings of previous research that propose a link between environmental conditions and phonology; the approach presented here tests these claims on a large language family, outlining both problems with previous approaches and shows that, at least for the Bantu family, the claims do not hold. For a robust analysis of the claims in general, more language families need to be considered in the future. In fact, developing a methodology that can estimate causal effects in cultural systems is the main aim of this paper, rather than supporting or rejecting the specific hypothesis about humidity and lexical tone. Indeed, based on the prior literature, we expect most of the variation to be explained by historical and geographic relations rather than a direct effect of humidity.

As outlined above, we estimate an predictive model for each of four different variables that have been suggested to be affected by humidity: lexical tones, number of phonological vowel distinctions, ratio of vowels to consonants in the segment inventory and ratio of vowels to consonants in core vocabulary (Everett, 2017; Everett et al., 2015; Maddieson, 2018). However, to simplify the descriptions of the concepts and models in Sections 3 and 4, we focus on explaining the model predicting the variable *tones* first. The other three models use the same methods and the results are summarised later in the paper.

The approach at hand uses Bayesian phylogenetic and Bayesian multilevel models because they are the most cutting-edge computational tools to infer and reconstruct phylogenies and ancestral states as well as enable the creation of complex, customisable statistical models and causal networks. It needs to be stressed that this is not a statement of value about the inherent usefulness of this framework regarding causal modelling in contrast to Frequentist approaches. In fact, this paper uses univariate Frequentist models in the simulations in Sections 3 and 3.1 because of their relative efficiency compared with Bayesian inference models.

## 2. Data

### 2.1. Empirical linguistic data

The number of tones for each language was obtained from the PHOIBLE database (Moran & McCloy, 2019). Missing data were filled in from the World Phonotactics Database (Donohue et al., 2013). A random sample of 20 other languages was obtained manually from language descriptions (see the Supporting Information). The number of vowels and consonants in the phoneme inventory of each language was also obtained from Moran and McCloy (2019). The vowel ratio in basic vocabulary was estimated from data in the Automated Similarity Judgement Program (ASJP version 20; CLLD, 2022) using a method similar to Everett (2017), except that we use the proportion of vowels used in the basic vocabulary compared with the total number of segments. Where there were multiple sources available for a language, the mean value was used.

### 2.2. Estimated ancestral states

The geographic–phylogenetic tree of Bantu was taken from Grollemund et al. (2015). This is a Bayesian phylogenetic reconstruction of the Bantu subfamily of languages which provides estimated ages and geographic locations for each ancestral node. These estimates were only available for the maximum clade credibility tree. Following the documentation, a reference year of 2000 CE was used.

Ancestral states for language data were estimated using Random-Walk Markov Chain Monte-Carlo Bayesian ancestral state reconstruction in BayesTraits (Meade & Pagel, 2022). A two-step procedure was used, first estimating a distribution of models from existing data, then estimating unknown values. Although the number of tones is a discrete measure, we used continuous ancestral state reconstruction since there were too many possible unique states for reliable estimate of a discrete state model. Proto-Bantu was fossilised as having two tones, based on traditional reconstructions from historical linguists (Nurse & Philippson, 2006; Stevick, 1969). A uniform prior from zero to 12 tones was used. The Bayesian process ran for a million iterations of burn-in and a million more iterations of sampling.

The same process was used to estimate the number of vowels and consonant segments in the phoneme inventory, and the vowel ratio in basic vocabulary. The prior biases were set according to the empirical distribution of all sources in PHOIBLE: a gamma distribution for the number of vowels (*k* = 3.3, *θ* = 0.3) and number of consonants; and a normal distribution for the vowel ratio (mean = 0.4659, SD = 0.0556). While the vowel ratio is technically bounded, the empirical distribution is far from either 0 or 1, making the bounds unreachable in practice. No nodes were fossilised for these alternative linguistic measures.

These processes resulted in a distribution of estimations for the linguistic measures at each node of the geo-phylo tree.

### 2.3. Humidity data

Humidity estimates for Sub-Saharan Africa for the last 10,000 years were taken from geophysical simulations (Davies-Barnard et al., 2017; Valdes et al., 2017). These estimate the Stephenson screen measurement of specific humidity (1.5 m above ground) for regularly spaced points in time and space across the world. A reference year of 1850 CE was used. To obtain estimates for specific points in time and space, the data were interpolated using a general additive model that predicted humidity given the year, latitude and longitude. A knot was specified for each unique year, latitude and longitude, and latitude and longitude had interactions by year. The model also included a tensor product smooth for all three variables. This effectively allows smooth interpolation between empirical observations in a three-dimensional space. In informal testing, the fit to the data was better for the general additive model (*R*^2^ = 0.81) than bilinear interpolation (*R*^2^ = 0.74).

### 2.4. Summary of data

The steps above resulted in a geo-phylo tree, where each leaf and ancestral node had estimates for their age, geographic location, humidity and the number of tones (and the alternative linguistic measures of vowels). This comprised data for 210 leaf languages for number of tones, 161 leaf languages for number of vowels and consonants, and 383 leaf languages for the ratio of vowels in basic vocabulary.

Tones had a weak phylogenetic signal (Pagel's *λ* = 0.35), while humidity had a very strong phylogenetic signal (Pagel's *λ* = 1.00, although we are not suggesting that humidity is inherited culturally). The number of vowels had a strong phylogenetic signal (Pagel's *λ* = 0.98), while the number of consonants had a slightly weaker phylogentic signal (Pagel's *λ* = 0.69). The ASJP vowel ratio had a very strong phylogenetic signal (Pagel's *λ* = 1.00)

All raw data, processing scripts and results are available in an online repository (https://github.com/seannyD/TonesClimateGeoPhylo_Public).

## 3. Biases in synchronic data produced by genealogical processes

Analysing datasets that stem from a process of genealogical evolution exhibits inherent biases that need to be accounted for in order to avoid biased results from statistical models. This section goes into detail about those biases, where they stem from and how to design models that can mitigate those effects.

**Figure 1.**
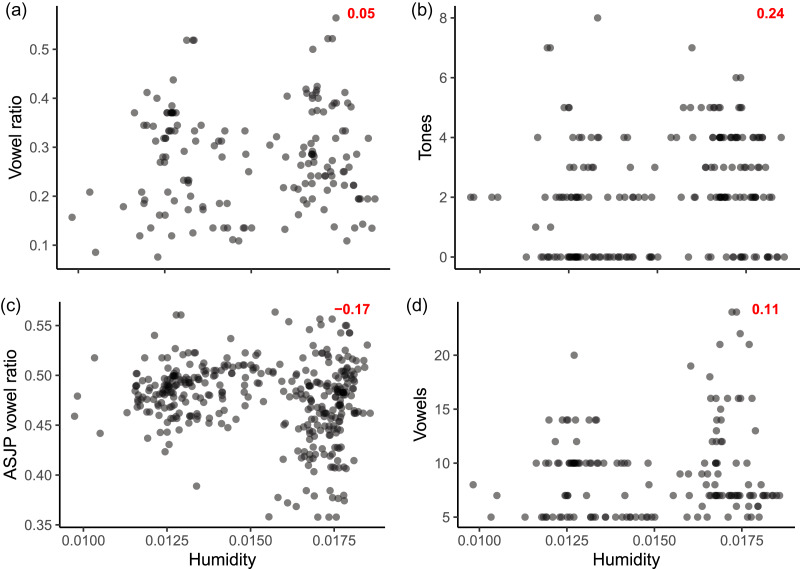
Scatter plots of all contemporary Bantu languages in the dataset by the four variables in question and humidity. Red figure: correlation coefficient of the two variables.

The contemporary Bantu languages show weak raw correlations between the phonological variables and humidity (see Figure 1). Without any adjustments through modelling choices, there are detectable correlational associations between humidity and the outcome variables in contemporary languages that appear to be statistically significant.

The correlation even survives controlling for phylogeny alone using two standard methods that test the strength of co-evolution of two variables (using BayesTraits; Meade, & Pagel, 2022). First, the discrete test (Pagel, 1994) applies to two binary variables that evolve down a phylogenetic tree. The method calculates a maximum-likelihood continuous-time Markov model of transitions between the four possible states of the two binary variables. An independent model, where both traits change independently is compared with a model where a change in one variable can influence the probability of change of the other. The tone variable was split into complex/non-complex tones (two or fewer vs. three or more, as in Maddieson, Bhattacharya, Smith, & Croft, 2011, fossilising tone at root) and the humidity variable was split into dry/humid regions (split at *h* = 0.0148, the mean humidity, and fossilising values into the tree). The discrete test suggested a significant relationship: languages are about twice as likely to gain tone than lose tone in high-humidity environments, and vice-versa in low-humidity environments (dependent model is preferred over an independent model, Bayes factor = 8.08). Secondly, a continuous random walk model (Pagel, 1999) treats the two variables as continuous and estimates a phylogenetic generalised least squares model using Browian motion. The root was fossilised as having two tones. This test indicates a weak but significant correlation between the number of tones and humidity (mean *r* = 0.07 [0.064, 0.074], Bayes factor compared with the model where correlation is zero with 100 stones and 1000 iterations per stone = 8.69). That is, analysing phylogenetic relations alone suggests that there is a relationship between tone and humidity. However, as we address in the following sections, the associations in the raw correlation and the phylogenetic tests are at least partially due to confounding effects of inheritance and borrowing. None of these simpler tests are able to account for borrowing or geographic autocorrelation. As we demonstrate below, a more general model is required to account for all of these effects together.

### 3.1. The null model of environmental effects

As a starting point of this investigation, we simulate the evolution in Bantu tones according to a null model with random changes. To do this, we iterate over the tree nodes and apply the following function:
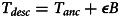


Here, the tones at the descendant node *T*_*desc*_ are equal to the ancestor node *T*_*anc*_ plus the product of a normally distributed error 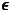 with standard deviation of 1 and the branch length *B*. The humidity information for every node is retained from the original dataset. According to this process, we simulate 100 random datasets and for each of these, we applied a simple Frequentist univariate regression to the leaf nodes (i.e. the extant languages) with tones as the outcome and humidity as the predictor variable. Thus, for each of these datasets, we obtain a slope of the predictor *humidity* along with a significance value at the level *α* = 0.05. In a dataset without significant bias, we would expect the type-I error rate for the predictor variable slope to be ~0.05, i.e. the slope in around five out of 100 datasets would be incorrectly significant.

**Figure 2.**
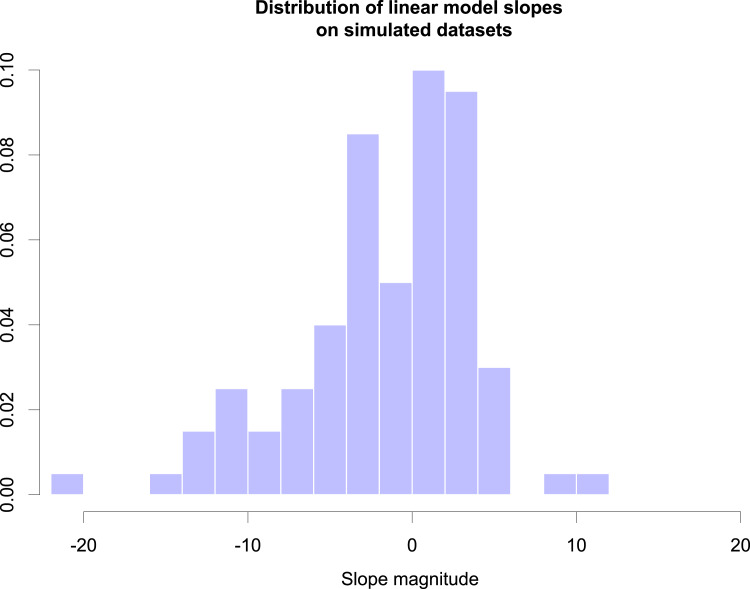
Histogram of the distribution of humidity predictor slopes for the simulated datasets under the null model.

The result of this simulation shows that the calculated slopes range widely from strongly negative to strongly positive slopes. Figure 2 shows a histogram of those predictor slopes.

Recall that the basis of those datasets is a null model in which humidity does *not* have any effect. Nevertheless we find that in 74 out of 100 datasets the univariate linear model detects a significant slope (either positive or negative) which is an extraordinarily high type-I error rate. This is indicative of a severe bias in the data generation process associated with the genealogical tree. However, what is it about this process that makes it exceptionally susceptible to these errors?

**Figure 3.**
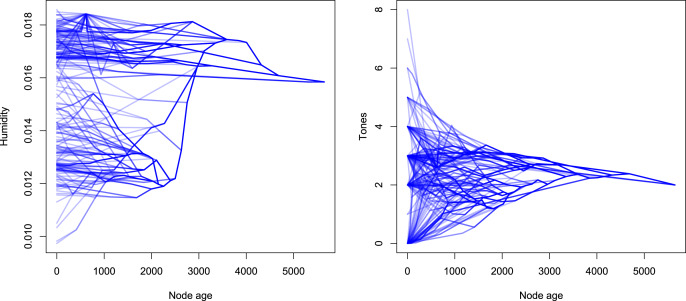
Evolution of humidity (left) and tones (right) along the Bantu language family tree.

To explore this issue further, we need to investigate the original dataset specifically regarding the distribution of humidity and tones along the structure of the phylogenetic model: Figure 3 is a visualisation of the diachronic development of *humidity* and *tones* along the nodes of the Bantu family tree. Here, each line segment represents a link between an ancestor node and a descendant node plotted against their respective humidity or tone values and the respective node age. The thickness of the lines is proportional to the number of terminal nodes that descend from this link.

**Figure 4.**
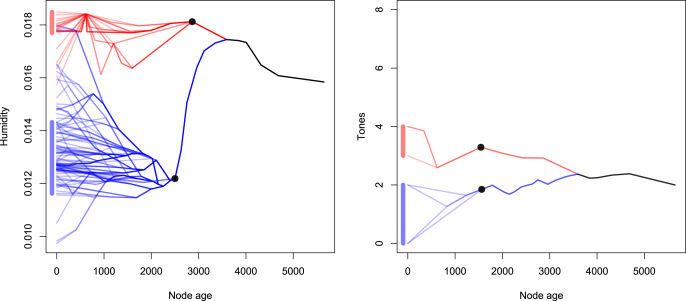
Two examples of clustering of decedents of two nodes in the phylomorpho space.

Especially salient is that in the plot of the humidity development, we find that over time, two clusters seem to form, one with high humidity values (> 0.016) and one with lower humidity values (< 0.016), the latter of which exhibits more variance. This clustering arises if there is one branch early in the tree that moves into more or less humid regions. We can demonstrate this phenomenon when we select two clusters and plot them separately. In Figure 4, we selected two representative clusters for each of the variables by selecting a node (represented by *black dots*) and plotting all descendant nodes from this point. Note that for this visualisation, different nodes were selected for tones and humidity.

In Figure 4, we can see that the two clusters differ considerably in the distribution of their terminal nodes. The line segments on the left of the graphs indicate the 80% highest density credible intervals for the distribution of the extant languages regarding humidity and tones. This means that, in the humidity graph, 80% of the languages descended from the blue lineage fall into a narrow range between approximately 0.0118 and 0.0142 humidity. A similar distribution can be observed for tones. This, however, is not necessarily problematic; to reveal the biasing effect of this distribution, we need to consider two major points.

Firstly, these clusters can arise owing to small changes early in the tree. When we consider the humidity graph in Figure 4, we see that the difference between the blue and red cluster is due to the fact that the ancestral community strongly and rapidly changed their environment between the most recent common ancestor of the red and blue lineage at about 3,500 BP and the ancestor node at 2,500 BP. After that, there are further no strong shifts. In effect, most of the contrast between the groups is due to a sudden change in environment by the ancestor node of the blue cluster. The environment in the history of this cluster is approximately homeostatic before 3,500 and after 2,500 BP. A similar, albeit weaker effect can be found for tones as well (see Figure 4).

The second driver of bias which builds upon this comes into play when the genealogically highly clustered tones and humidity interact. Specifically when clusters align or partially align (i.e. *many tones – high humidity*, or *many tones – low humidity* and vice versa), strong positive and negative correlations are easily created. Further, owing to the cluster contrasts resulting from changes early in the tree, smaller variations early on can result in strong founder effects for subsequent nodes. This is the reason why we find alternatingly strong positive or negative effects, despite the simulation datasets being generated by a null model.

### 3.2. Node importance bias

To define even more precisely the driving factor of this process, we can investigate node importances, specifically, which nodes have the strongest impact on the outcome. Put differently: in which nodes are small variations likely to cause a bias in the synchronic data?

**Figure 5.**
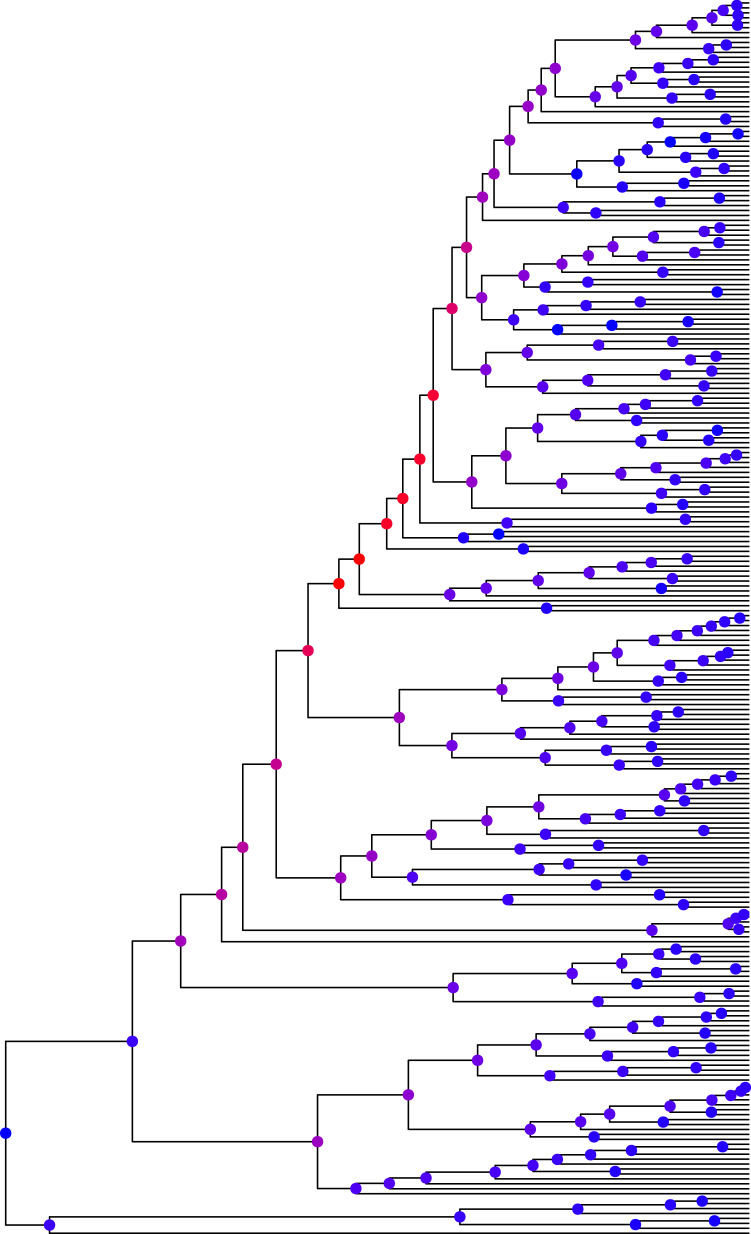
Bantu family tree with node importance ranging from blue (less important) to red (more important).

To calculate which nodes are most influential in shaping the outcome, we create a new dataset for each node in the tree where a fixed value is added to the tone values of all descendants of this node (in this case, we add 1). This means that we create a large number of datasets that each differ in the tone values of one lineage. In a second step, we take the predictor slope of *humidity* on *tones* in the new dataset and calculate the absolute distance to the predictor slope in the original, unaltered, dataset. Subsequently, we obtain for every dataset a value of how strongly changing the tone value of a certain node changes the calculated predictor slope. This results in a per-node value of importance since nodes that are more influential cause a greater shift in the regression slope. Figure 5 shows the Bantu family tree with every node coloured according to its importance.

We can observe that the most important nodes are those that (1) are located somewhat early in the tree and that (2) have many, closely clustered descendants. For this tree, eight out of 499 nodes can be viewed as highly important which means that around 1.6% of the observations in the dataset determine whether we see a positive/negative/no correlation between humidity and tones synchronically. we expect that such biases exist in other language families and across other phonological features. This is a major problem for correlational studies on synchronic data, especially for studies which only control at the level of language family rather than a full phylogeny, since this example demonstrates that intra-family biases can lead to distorted findings.

## 4. Modelling typological evolution

Taking into account the findings concerning genealogical bias in Section 3, we need to design a scientific model investigating environmental effects on phonology that is unaffected by this bias. The following section goes into detail about the considerations of the scientific model we use for analysing this relationship.

### 4.1. Accounting for genealogical bias

Given the aforementioned issues of using synchronic data that are the result of a genealogical process, we aim for a diachronic model to remove these biases. Rather than modelling the synchronic relationship between *humidity* and *tones*, we model the difference in tones between an ancestor node and a descendant node.

If *humidity* indeed had an influence on the diachronic development of *tones*, we would expect to see that languages in more humid environments tend to develop more tones. In other words, if this conjecture is true, then we would see that languages located in more humid climates would tend to acquire more tones over time than languages in more arid environments would. This focus on changes between nodes removes the genealogical bias since the clusters that form in synchronic data are not present.

### 4.2. Causal relationships

**Figure 6.**
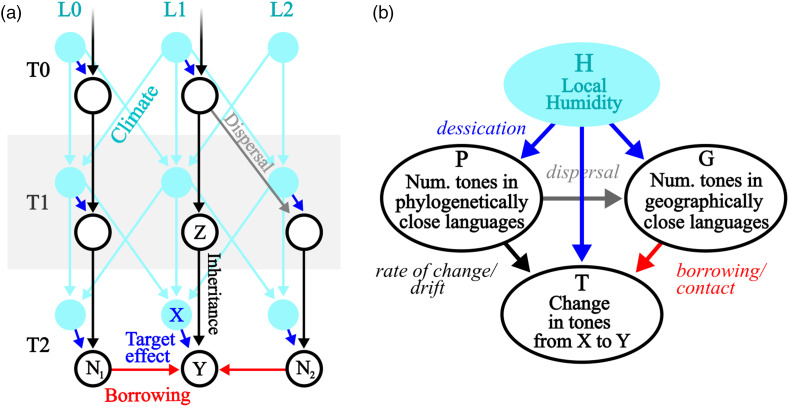
(a) Causal relationships between cultural variables (white circles) at different points in time (T0, T1, T2) and the environment (blue circles) at different geographic locations (L0, L1, L2) via inheritance (black and grey lines, e.g. from Z to Y), borrowing (red lines, e.g. from *N*_1_ to Y), and a target effect of the environment on the cultural variable (blue line, e.g. X to Y). (b) A simplification of the graph focused on node Y, with the environmental effects collapsed into one node.

There are multiple causal relationships associated with humidity and tones. Here, we use tools from causal inference to map out these causal relationships in order to arrive at the form of our statistical model. Figure 6a shows some complex relationships between hypothetical cultures and climates as a causal network (or ‘Bayesian network’; see Pearl, 1985). Imagine a group of cultures that disperses over time (T0, T1, T2) and moves across a geographic space (locations L0, L1, L2). We are interested in estimating the effect of humidity *X* on the number of tones in the language spoken by culture *Y*. However, the number of tones in *Y* is also affected by cultural inheritance from an ancestor culture *Z* and borrowing from geographically proximal cultures *N*_1_ and *N*_2_. These associations need to be partialled out of the effect of *X* on *Y*. Furthermore, the local humidity is part of a complex network of climatic dependencies in time and space. This creates formal confounds for estimating the effect of *X* on *Y*, for example non-causal pathways via climatic dependencies and inheritance or borrowing. Therefore, the geospatial dependencies also need to be controlled for in order to accurately estimate the effect of humidity on the number of tones.

In order to take account of these multiple confounds in a statistical model, we collapse the causal graph to focus on local effects (Figure 6b). In this causal model, *T* is the change in number of tones between an ancestor node and a descendant node (see Section 4.3.1). We model the change in tones from the ancestor node to the descendant node to be able to model the relative change with a Gaussian outcome rather than the absolute number of tones. This also removes the ancestor node in the predictor (mathematically, the two formulations *Tones*_*descendant*_ − *Tones*_*ancestor*_ ≈ *predictors* and *Tones*_*descendant*_ ≈ *Tones*_*ancestor*_ + *predictors* are equivalent).

The effect of the number of tones in phylogenetically close languages on tone change (*P* → *T*) represents confounds associated with genealogical relationships beyond the effects that are already controlled for (see Section 4.1). These residual effects include but are not limited to:

- *Rate of change* – some lineages might exhibit a higher propensity to lose/acquire tones, i.e. a higher change rate.
- *Drift* – some closely related languages might develop more tones independently (after the split from a common ancestor), but owing to a common cause inherited from their ancestor. In effect, if tones are not inherited from their ancestor, but a certain phonological situation is, that leads to the development of tones in the future.

It is calculated in the model as the average tones in a language's closest relatives or, in the second model, as a Gaussian Process (see discussion in Section 4.3.4).

Further, the effect of geographically close languages on the number of tone change (*G* → *T* ) represents geographical confounds associated with the (non-)acquisition of tones from geographically proximate groups. These influences include but are not limited to:

- *Intra-family contact* – borrowing of tones from related languages that are geograpically close.
- *Contact* – borrowing of tones from unrelated languages that are geographically close.
- *Isolation* – effects from a lack of contact with other groups.

These effects can cause a change in tones since, for example, languages may increase their number of tones through borrowing when surrounded by languages with many tones. If, conversely, a language is surrounded by languages without tones, no tones can subsequently be borrowed. *G* is calculated in the model as the average tones in a language's vicinity or, in the second model, as a Gaussian Process (see discussion in Section 4.3.4). In the current work, we calculate this based only on Bantu languages in our sample. While there are neighbouring languages from other families that may have tone, we leave this for future work, since we do not have reliable data on the geo-phylo histories of those languages.

The *Local Humidity* node represents the climate network of humidity across time and space. If humidity influences tones, then we would assume it to do so on every level. Higher humidity would increase the change in tones between ancestor and descendant nodes but it would also increase the number of tones in the vicinity of a language and the tones in its related languages. In the main model only the effect of *H* → *T* is modelled. In order to model the assumption that the effect of humidity on language takes place at some point between the ancestor node and the child node, the model measures *H* as the average humidity of the location at the ancestor node and the descendant node. That is, the time points of changes from one humidity level to another between the ancestor and the descendant are unknown, but we assume that they average out across the sample. We assume consistency (Rehkopf, Glymour, & Osypuk, 2016) and temporal stability: that the effect of humidity is not a composite measure and has the same effect in any situation. This might be violated by, for example, modification of ambient humidity by modern air conditioning, though this would only have very recent effects on the data.

The statistical model predicts *T*. We make a causal Markov assumption, meaning that three effects are not represented in the statistical model: *H* → *P*, *H* → *G* and *P* → *G*. This is because these effects are not directly causally relevant for predicting *T*.

We also assume that the causal network above is complete and that no major unmeasured confounds exist that causally influence both change in tones and humidity. This may not be realistic, but is a simplifying assumption for the sake of this study. Potential candidates for additional confounding effects include, for example, the effect of population size on rates of cultural change (Greenhill, Hua, Welsh, Schneemann, & Bromham, 2018), or the relationship between climate and population dispersal (Honkola et al., 2013).

Finally, we assume that specific humidity is the only climatic variable that affects tone. While some studies of linguistic adaptation use temperature as their environmental variable, Everett (2017, p.4) explains that ‘temperature is an (imperfect) proxy for specific humidity because air at colder temperatures can “hold” less water’.

### 4.3. The inference models

For the computational modelling of the causal model outlined in Section 4.2, we employ the probabilistic programming language STAN (Stan Development Team, 2022) to design two different Bayesian models (named *LIN* and *GP*) which differ in their handling of the geography and phylogeny variables (see Section 4.3.4). The following sections go into detail about the individual components of the models and what parameters are used, while at the end, the full model equations are given in Section 4.3.5.

#### 4.3.1. The model core

The cores of both models are the outcome and the main linear model:
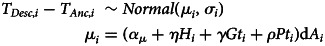


Here, the change from the number of tones at the ancestor node *T*_*Anc*_ to that of the descendant node *T*_*Desc*_ is modelled as a normal outcome with mean *μ* and variance *σ*. The mean for each observation *i* is calculated as the sum of the population intercept 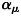, and the products of the linear coefficients *η*, *γ*, *ρ*, the respective humidity *H* and tones in geographically (*Gt*) and phylogenetically (*Pt*) close languages at observation *i*. This sum is multiplied by the temporal distance d*A* between the ancestor node and the descendant node, measured in units of 1000 years. This last term serves as a normalising factor which normalises the effects to effects per 1000 years. Doing this is important as we expect that the effects scale with time, i.e. languages that only recently split from their ancestor are expected to have less time to undergo any changes. This prevents the model from finding a null effect for languages with a small time distance from their respective ancestor.

#### 4.3.2. Modelling time distance variance increases

The mean *μ*, however, is not the only part of the model affected by time distance between ancestor and descendant node. We also need to account for the possibility that with increasing time difference between the node pair, variance in the observation also increases.

On theoretical grounds, we can make such an assumption since we expect the unmeasured effects to compile over time such that with increasing temporal distance, the outcome is affected by increasing variance. In practical terms, the more time difference there is between the observation at the descendant node and the ancestor node, the longer other effects outside of this model can act on the process and increase the variance. The equation for this part of the model is as follows:
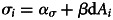


Here, for every datapoint *i*, the model calculates the individual variance to be equal to a global baseline variance 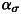 plus the product of the coefficient *β* with the temporal distance d*A* between the node pair. Note that *β* is inferred as an unconstrained variable with a standard normal prior which results in an unconstrained slope. This relaxes the assumption about whether a variance increase exists in the data; the specific shape of a potential difference in variance dependant on d*A* is therefore inferred from the data.

Doing this prompts the model to assume the possibility that more distant descendant–ancestor node pairs fit the data less well. This again is intended to prevent the model from finding a null effect solely because of the unequal distribution of variance along the temporal axis. Since *β* is unconstrained, however, if there is not enough evidence for this in the data, the model will not assume such a variance increase.

#### 4.3.3. Modelling uncertainty in reconstruction

As discussed in Section 2, the information about the tones at each node is drawn from a Bayesian phylogenetic model and the interior nodes of the tree therefore are reconstructions. However, since the tone values for interior nodes are posterior draws, we need to account for reconstruction uncertainty for these nodes. Therefore, we use this mechanism in the model to incorporate this reconstruction uncertainty:
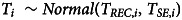


Each interior node's tone value *T* is drawn from a normal distribution with a mean equal to the posterior mean of that node in the phylogenetic inference model *T*_*REC*_ and a variance equal to the standard error of this posterior distribution *T*_*SE*_. Doing this ensures that the model recognises different degrees of uncertainty in the tone values of interior nodes.

#### 4.3.4. Modelling contact and phylogenetic effects

The treatment of the potentially confounding variables of *phylogeny* and *geography* is where the two models (model LIN and model GP) differ. There are two ways of incorporating both variables in such models, each with their own drawbacks.

In *model LIN*, the geographical control is, simplified, the average tone count in the geographically/phylogenetically close languages. *Model LIN* is therefore a model with fewer variables that is additionally sensitive to which other languages are contemporary to the node in question. The drawback here is that it contains stronger assumptions about what constitutes geographic and phylogenetic closeness. *Model GP* foregoes these assumptions and models geography and phylogeny as Gaussian processes based on pairwise distance matrices. This makes the model better at modelling gradation in these variables, but at the same time it strongly increases the model complexity and, owing to the constraints of the kernel functions, cannot account for which languages are contemporaneous.

**Figure 7.**
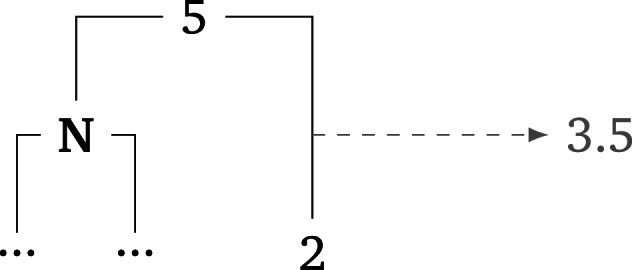
Visualization of the interpolation of tones in neighbouring lineages at a given split time.

The specific implementation of these two different architectures is as follows. For *Model LIN*, the geography variable represents the per-node mean and variance of the number of tones in the languages that are contemporaneous to the node and less than 500 km away. Since the tone data are drawn from a phylogenetic tree where the splits occur at different times, the tones in the contemporaneous lineages are linearly interpolated according to the following function:
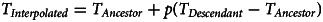
where *p* is the proportional time point where the node in question is temporally located. For example, let *N* be a node for which the contemporaneous tone value of a neighbouring lineage is calculated. Assume that *N* occurs temporally exactly half way between the ancestor (five tones) and descendant (two tones) nodes of this neighbouring lineage. Figure 7 visualises this example.

Here, the interpolated tone value of the neighbouring lineage at the time of node *N* is 3.5 since 5 + 0.5(2 − 5) = 3.5.

**Figure A1.**
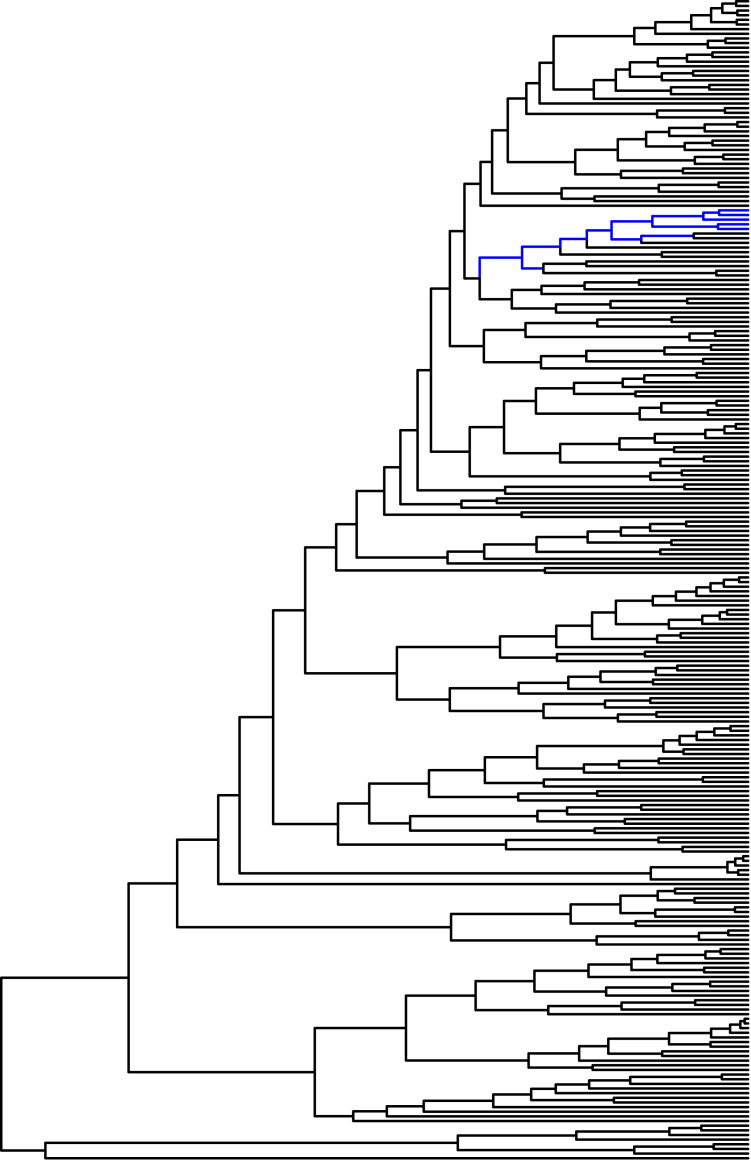
An example of the nodes with distance 0.02 from a selected node, highlighted in blue.

The phylogeny variable is devised in much the same way where for every node, the mean and standard deviation were calculated of all phylogenetically close nodes with a cophenetic distance of less than 0.02, which corresponds to a small phylum (see Figure A1 in the Appendix for an illustrative example). Note that, here, the nodes were not filtered for contemporaneity in accordance with the intended purpose of this variable as representing residual phylogenetic influences of a lineage (see Section 4.2).

Owing to how the two variables are designed, there is variance in the tone means, which is accounted for in the model:
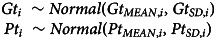


Here, we estimate the average tones in the geographically/phylogenetically close languages (*Gt*, *Pt*) of node *i* as a variable drawn from a normal distribution with mean equal to the tone mean of the observed data (*Gt*_*MEAN*_, *Pt*_*MEAN*_) and variance equal to the standard deviation of the observed data (*Gt*_*SD*_, *Pt*_*SD*_). Doing this accounts for the inherent variation in the data aiming to incorporate the variance in the distribution of tones in close languages; taking only the mean could obscure important patterns.

In *Model GP*, the geography and phylogeny variables represent residual expected geographical/phylogenetic similarity between individual languages. The model architecture is built to infer these similarities from Gaussian processes on the basis of geographical and phylogenetic distance (as detailed above). The model structure is as follows:
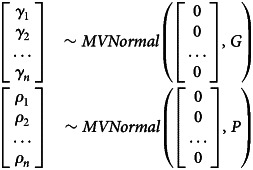


Here, the expected confounds for every node (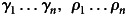) are drawn from multinormal distributions with means of 0 and covariance matrices *G* and *P* as the kernel function.

The covariance matrix for the geographic confound (*G*) is a variation of a squared quadratic kernel whereas *P* represents a variation of the Ornstein–Uhlenbeck kernel:
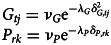


Here, *δ* = |*x* − *x*′| where *x* − *x*′ is the phylogenetic/geographical distance between two languages in a distance matrix. This modelling approach is similar to what is described in McElreath (2018, chapter 14.5). As mentioned before, the geographical variable in this implementation cannot account for which languages are contemporaneous. This is due to the constraints of the Gaussian process kernels which can only process positive definite matrices, which requires all languages to be simultaneously present.

#### 4.3.5. Full model summaries

After the detailed discussion of the model components, this section shows the models in full with all components put together. The first model (*Model LIN*) is a model where the effects of phylogeny and geography are modelled linearly. Recall that the model structure indicated here refers to the model using analysing the variable *tones*. The models for all four variables are equivalent with the exception that the priors used for the modelling of the vowel ratio variables are more informative: for those parameters that in the following equations show a prior of *Normal*(0, 1), we used *Normal*(0, 0.5) instead in these cases and instead of *Exponential*(1), we used *Exponential*(5).Equation of model LIN
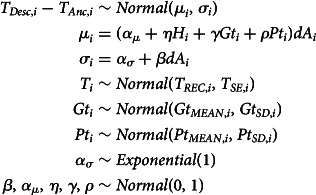


The second model (*Model GP*) is a variation of the first in which the effects of phylogeny and geography are modelled using Gaussian processes:Equation of model GP
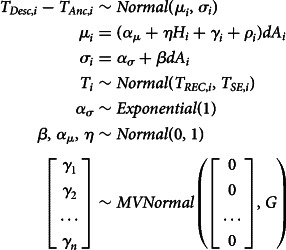

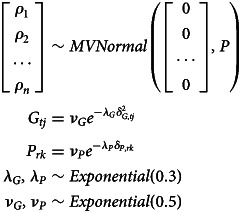


In all models, the input variables were *z*-scaled (not centred) such that one unit in the model corresponds to one standard deviation in the data.

### 4.4. Model comparison

Since both ways of including geographical/phylogenetic confounds have advantages and disadvantages, we compared the two model approaches along with the individual confounds. For this, we used the R-package *loo* (Vehtari et al., 2022) to calculate the pairwise expected log predictive density (ELPD) for each model to obtain the most predictive model given the individual parameters. Table 1 shows the variables and model type that achieved the best scores.

**Table 1.** Variables in best models

Model	Humidity	Geography	Phylogeny	GP
Tones	×	×		
Vowels	×	×		
Vowel ratio	×	×	×	×
ASJP vowel ratio	×	×	×	

Here, the checks indicate a control variable being included and *GP* indicates whether the model uses the linear or the Gaussian Process approach. We can see that in the models for the two vowel ratio models, phylogeny is more predictive than in the raw tones or raw vowels models. Further, the only Gaussian Process model is that of *vowel ratio*. The detailed coefficients of the model comparison analysis can be found in the appendix. Going forward, we only report the results from these models in the following sections, meaning that whenever we refer to the *tones* model, we refer to the model with the specifications indicated in Table 1.

## 5. Results

**Figure 8.**
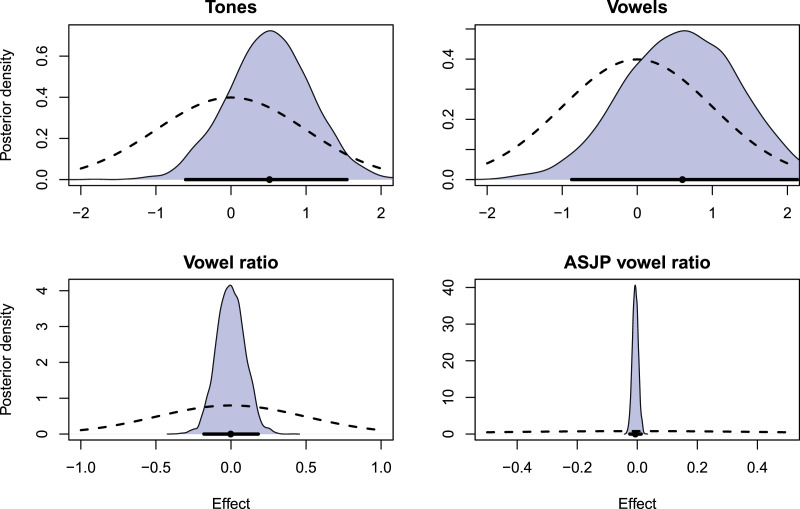
Posterior distribution of conditional effects of *humidity* on the four datasets. Blue, density of posterior samples; dashed curve, prior density; black line segment, 95% highest density interval; black dot, posterior mean.

Figure 8 shows the distributions of posterior effects in the model, specifically the slope of the temporal variance model, humidity, and the geographical and phylogenetic confounds.

In all cases, there is no significant effect of *humidity* on the outcome as the posterior compatibility intervals either cover a wide effect range (in the case of the tones and vowels model) or show a precise estimate of a null effect (in the vowel ratio models). Further, the Bayes factors (more concretely, the Savage–Dickey density ratios) for the parameters are low (see Table 2), meaning that there is insufficient support for these parameters being different from 0. The other posterior model parameters, not directly part of the research question can be found in Table A1 in the Appendix.

**Table 2.** Savage–Dickey density ratios for the *humidity* effects across the models

Model	Bayes factor
Tones	0.863
Vowels	1.06
Vowel ratio	0.185
ASJP vowel ratio	0.11

## 6. Discussion

The model results show that there is no discernible effect of humidity on any of the phonological variables as proposed in previous studies. In addition, for some of the variables (e.g. tones and vowels), the posterior distribution is only weakly more informative than the prior, suggesting that there is little signal in the data to begin with. The analysis has shown that the environmental effect of humidity found in datasets with contemporary data does not hold when tested with a diachronic causal model that accounts for historical relations between languages, changes in related languages, historical changes in humidity and language contact.

One of the primary reasons for this is that, as demonstrated in detail in Section 3, the tree structure of language evolution lends itself to clustering of similar properties and non-linguistic variables even if their mutual association is accidental. Few important ancestor nodes higher up in the tree can determine or remove such associations as changes in those nodes propagate to multiple contemporary making an association seem more robust synchronically than it is diachronically. This demonstrates the necessity for studies on environmental effects to analyse the datasets diachronically, taking into account the shape of the change-generating process (tree-like evolution in this case).

It is possible that an alternative model structure would have revealed different results. For example, modelling different rates of gain and loss of tone as the critical nodes in the causal graph, rather than the actual number of tones (see Moran, 2016). This would avoid assuming a linear, bidirectional relationship. However, there are remaining complexities to solve before this is feasible, including ensuring that the model does not estimate a number of tones outside the range of possible values, and dealing with the increased model complexity. In any case, we think that it is unlikely that an effect would be absent in the simpler model but present in the more complex one.

It is also possible that an alternative phylogenetic tree of Bantu would yield a different result (e.g. Koile, Greenhill, Blasi, Bouckaert, & Gray, 2022). However, the differences between the two phylogenies are relatively minor in terms of humidity estimates at critical nodes. Similarly, the results might be different for an analysis of a different language family such as Sino-Tibetan (Sagart et al., 2019; H. Zhang, Ji, Pagel, & Mace, 2020), since the processes of language change can vary by language family (Dunn, Greenhill, Levinson, & Gray, 2011). However, Bantu is one of the largest families with an estimated geo-phylo tree and with a large variation in humidity. Additionally, even if an effect was found, the theory would need to account for why the causal effect is seen in one family but not another.

Finally, the ability of the inferential model to detect a real effect depends on an unbiased process for the ancestral state reconstruction of the linguistic variables. However, since the ancestral state reconstruction process used here does not incorporate information on humidity, it might bias the data in favour of the inheritance component of the model and against the humidity component. The size of this bias could be determined in future studies using artificial data that has been simulated with an explicit effect of humidity. Indeed, an alternative approach would be to simulate the whole process including phylogeny, borrowing and humidity, and estimate the strength of effects using a generative framework (Kandler & Powell, 2018), bypassing the need for ancestral state reconstruction. In this case, a parameterised generative model would be required, and the inferential model in the current study could serve as a starting point. A further limitation here, which is inherent to all deep-time diachronic studies that involve phylogenetic reconstruction, is that the data for the interior nodes are reconstructions rather than observed data points. Although countermeasures were taken to mitigate this issue (see Section 4.3.3), the analysis depends on the assumption that the ancestral states reconstructions are representative approximations of the linguistic variables of interior nodes.

## 7. Conclusion

We presented a study which showed how the use of causal inference can help guide studies which seek to investigate the relationship between linguistic features and language-external phenomena. We used historical data and ancestral state reconstruction to represent the full history of the relationships. The flexibility of the regression framework allowed us to control for historical and geographical confounds. The simultaneous estimation of several causal effects also moves the focus away from trying to explain the variation in a single target variable towards analysing a wider network of interactions.

No significant effect of humidity on linguistic features could be detected. As such, the current study contributes to a growing body of research based on multiple datasets and using multiple methods which have found no supporting evidence for an effect of humidity on lexical tone. Still, we note that the study of large-scale, cross-cultural relationships have developed methods and driven the need for more advanced data and estimation tools. We hope that the current contribution combining causal inference and historical climate models can be useful for future studies of cultural evolution.

## Supporting information

Hartmann et al. supplementary material 1 Hartmann et al. supplementary material

Hartmann et al. supplementary material 2 Hartmann et al. supplementary material

Hartmann et al. supplementary material 3 Hartmann et al. supplementary material

Hartmann et al. supplementary material 4 Hartmann et al. supplementary material

## Data Availability

All data and processing scripts are available at https://github.com/seannyD/TonesClimateGeoPhylo_Public or https://doi.org/10.5281/zenodo.10458491.

## Supplementary material

To view supplementary material for this article, please visit https://doi.org/10.1017/ehs.2023.33

## Research transparency and reproducibility

All data and processing scripts are available at https://github.com/seannyD/TonesClimateGeoPhylo_Public or https://doi.org/10.5281/zenodo.10458491.

## Appendix

Tables A2–A5 show the associated ELPD difference values between the models (see Section 4.4). These were obtained through bottom-up model fitting, determining the best model by iteratively adding terms. Note that in the analyses for variables *vowelratio* and *vowels (ASJP)*, convergence issues occurred with some GP models which were subsequently discarded. This can occur when, for some variables, the terms that are modelled as Gaussian Processes are highly correlated.

**Table A1.** Posterior summary of the variables by model. The parameter names correspond to the parameters outlined in the model equations in Section 4.3

Variable	Model	Mean	Lower CI	Upper CI
*α*	Vowels	0.36	−1.03	1.78
*α*	ASJP vowel ratio	−0.01	−0.05	0.04
*α*	Tones	−0.22	−1.11	0.82
*α*	Vowel ratio	−0.03	−0.61	0.47
*η*	Vowels	0.60	−0.87	2.13
*η*	ASJP vowel ratio	−0.01	−0.02	0.01
*η*	Tones	0.51	−0.60	1.55
*η*	Vowel ratio	−0.00	−0.18	0.18
*γ*	Vowels	−0.09	−0.27	0.10
*γ*	ASJP vowel ratio	0.03	−0.06	0.11
*γ*	Tones	−0.12	−0.40	0.17
*ρ*	ASJP vowel ratio	−0.00	−0.03	0.02
	Vowels	0.82	0.62	1.02
	ASJP vowel ratio	0.01	0.00	0.01
	Tones	0.75	0.61	0.91
	Vowel ratio	0.01	0.00	0.01
*β*	Vowels	1.65	1.22	2.09
*β*	ASJP vowel ratio	0.02	0.02	0.02
*β*	Tones	0.34	0.06	0.62
*β*	Vowel ratio	0.01	−0.00	0.02
*ν*_*G*_	Vowel ratio	0.10	0.00	0.49
*ν*_*P*_	Vowel ratio	0.11	0.00	0.56
*λ*_*G*_	Vowel ratio	1.44	0.00	6.84
*λ*_*P*_	Vowel ratio	0.86	0.00	4.66

**Table A2.** loo comparison with expected log predictive density (ELPD) for analysis of variable *tones*.

Model	Difference in ELPD	Standard error
Humidity	0.00	0.00
Full	−0.37	2.82
Humidity + geography	−1.76	1.55
Full (GP)	−3.20	1.31
Humidity + geography (GP)	−4.20	3.70

**Table A3.** *loo* comparison with ELPD for analysis of variable *vowelratio*.

Model	difference in ELPD	standard error
Full (GP)	0.00	0.00
Humidity	−274.94	15.31
Full	−276.28	15.39
Humidity + geography	−276.41	15.28

**Table A4.** *loo* comparison with ELPD for analysis of variable *vowels*.

Model	difference in ELPD	standard error
Humidity + geography	0.00	0.00
Full	−1.08	1.88
Humidity	−3.15	1.94
Full (GP)	−320.35	29.15
Humidity + geography (GP)	−325.85	29.35

**Table A5.** *loo* comparison with ELPD for analysis of variable *vowels (ASJP)*.

Model	difference in ELPD	standard error
Humidity	0.00	0.00
Full	−1.82	1.77
Humidity + geography	−2.04	1.66
