# Supplementary figures and images for: Investigating environmental effects on phonology using diachronic models
Source: Evol Hum Sci. 2024 Jan 3;6:e8. doi: 10.1017/ehs.2023.33 (PMC10955398; doi:10.1017/ehs.2023.33)

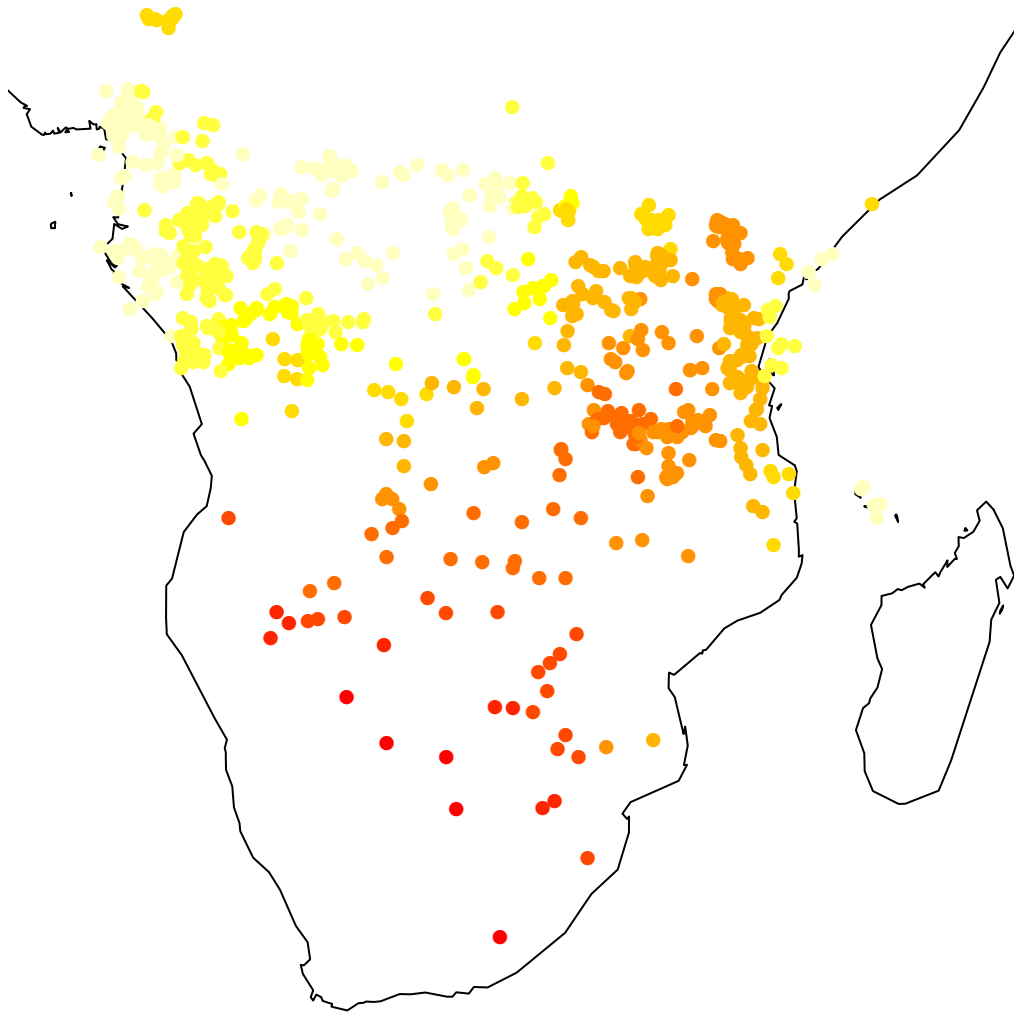

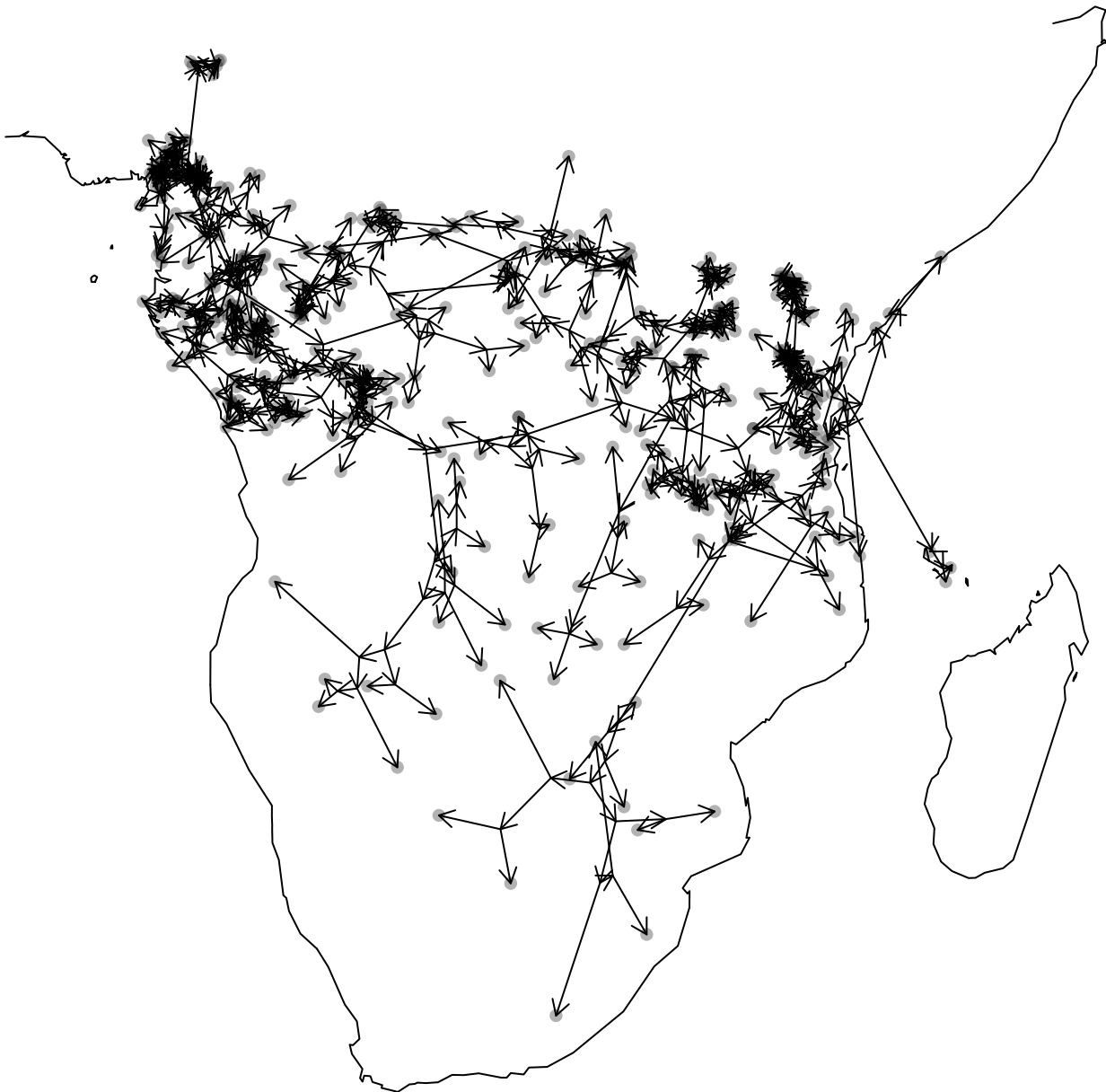



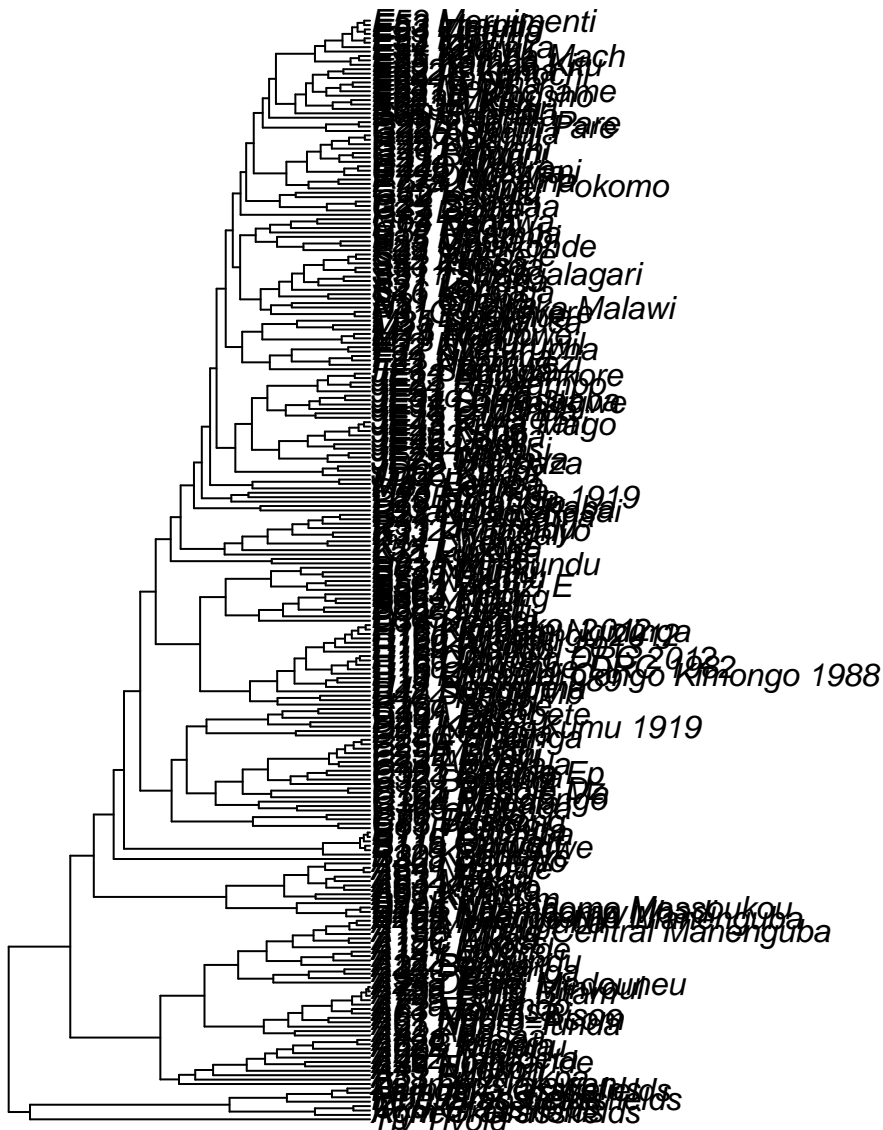

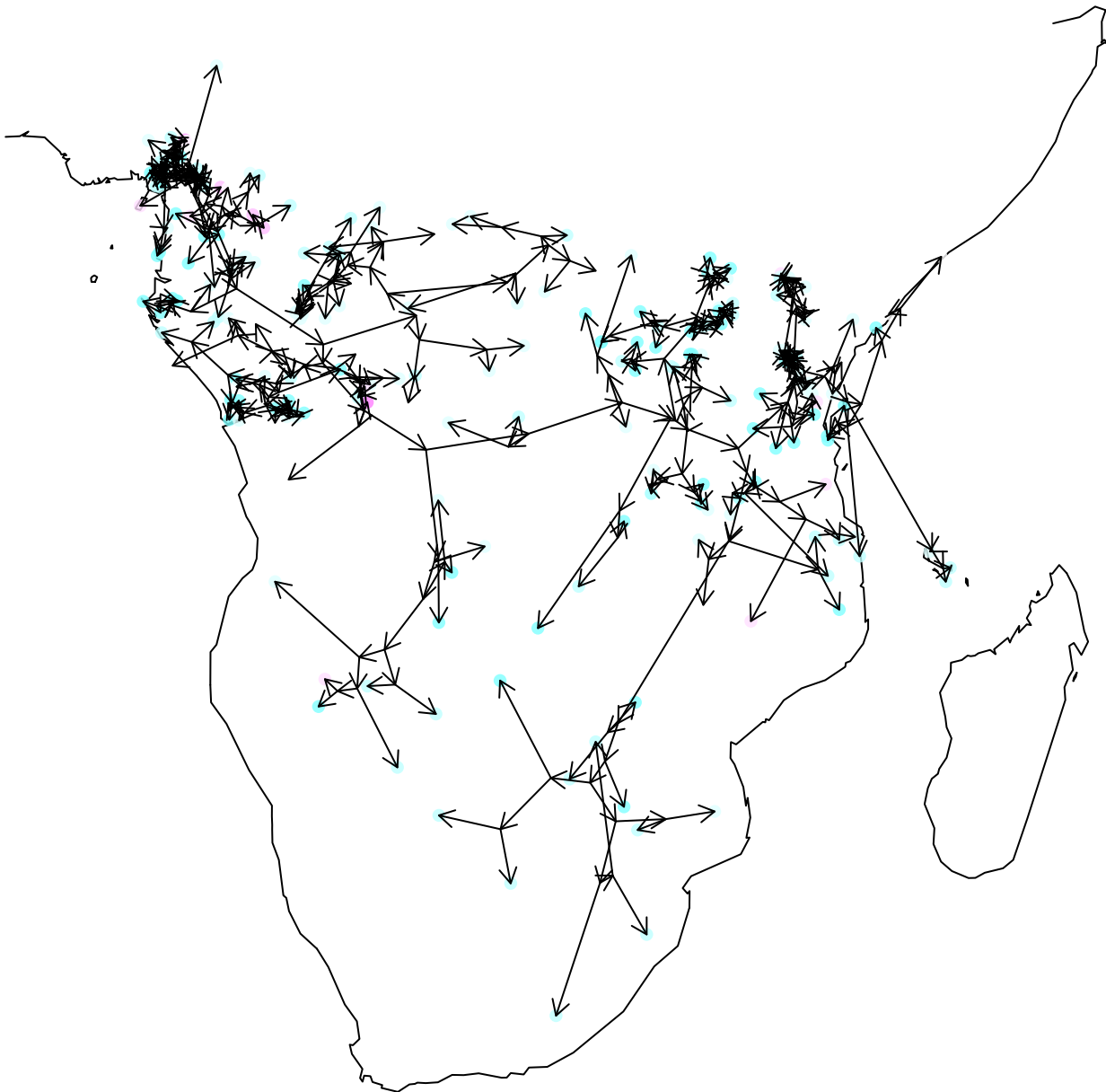

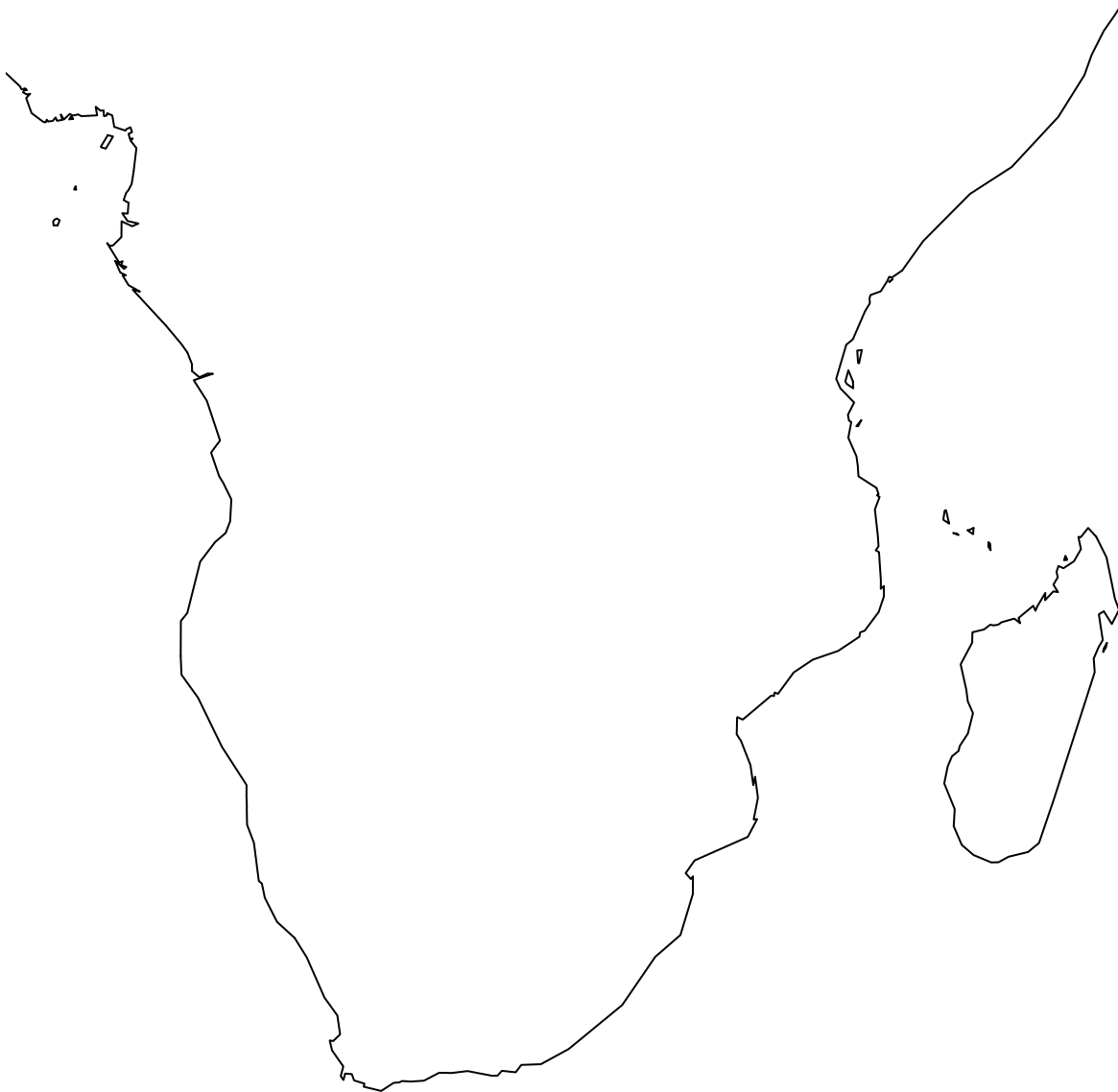

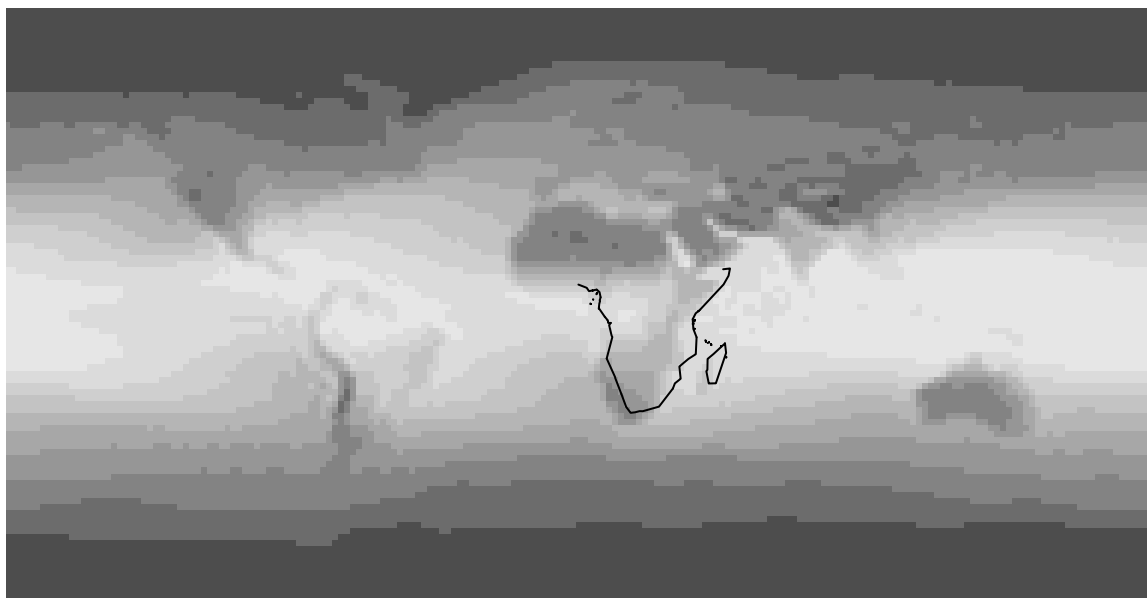

Supplement: Hartmann et al. supplementary material 4 — Hartmann et al. supplementary material [file S2513843X23000336sup004.zip › TonesClimateGeoPhylo_Public-1.0/analysis/Rplots.pdf]

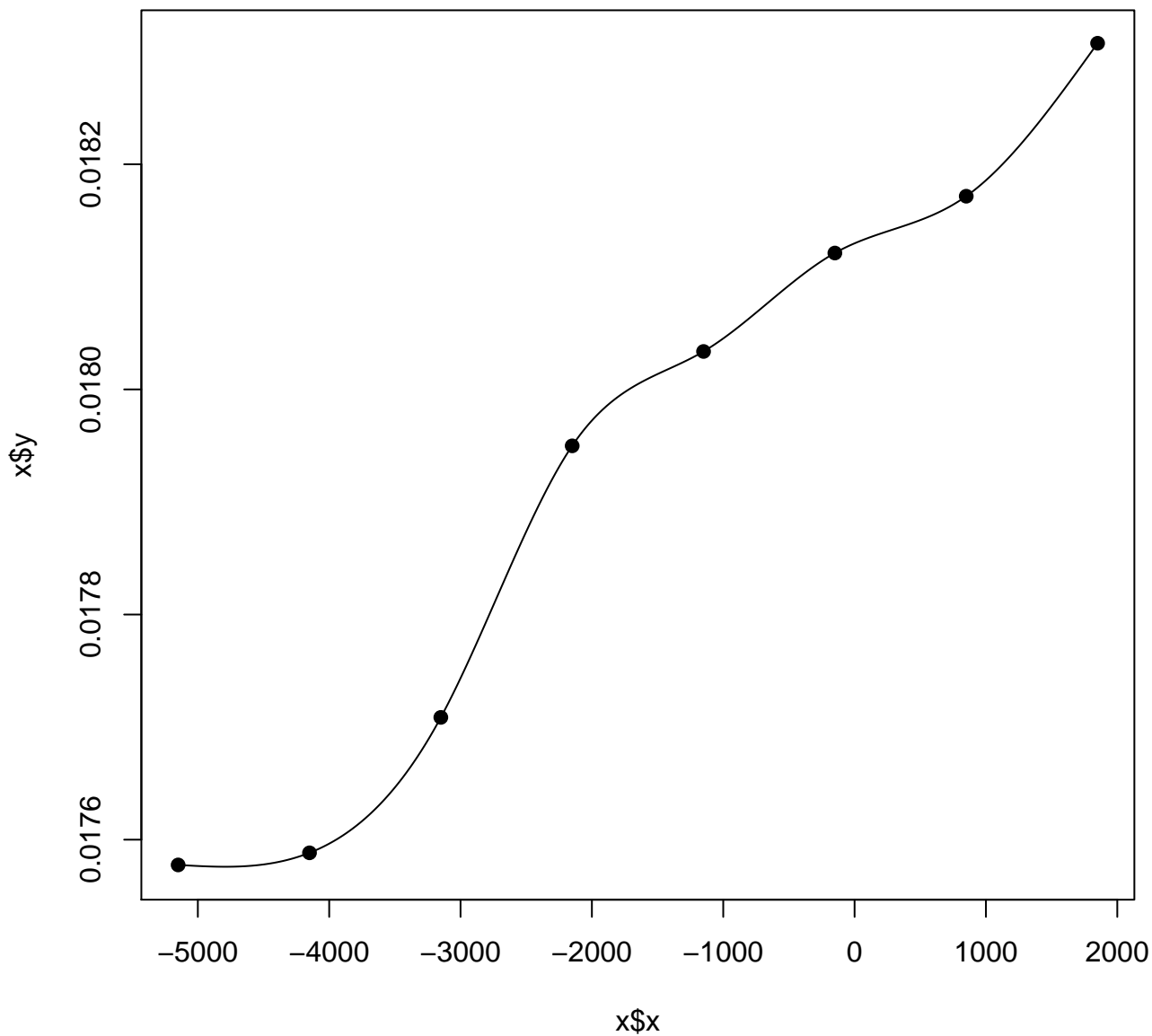

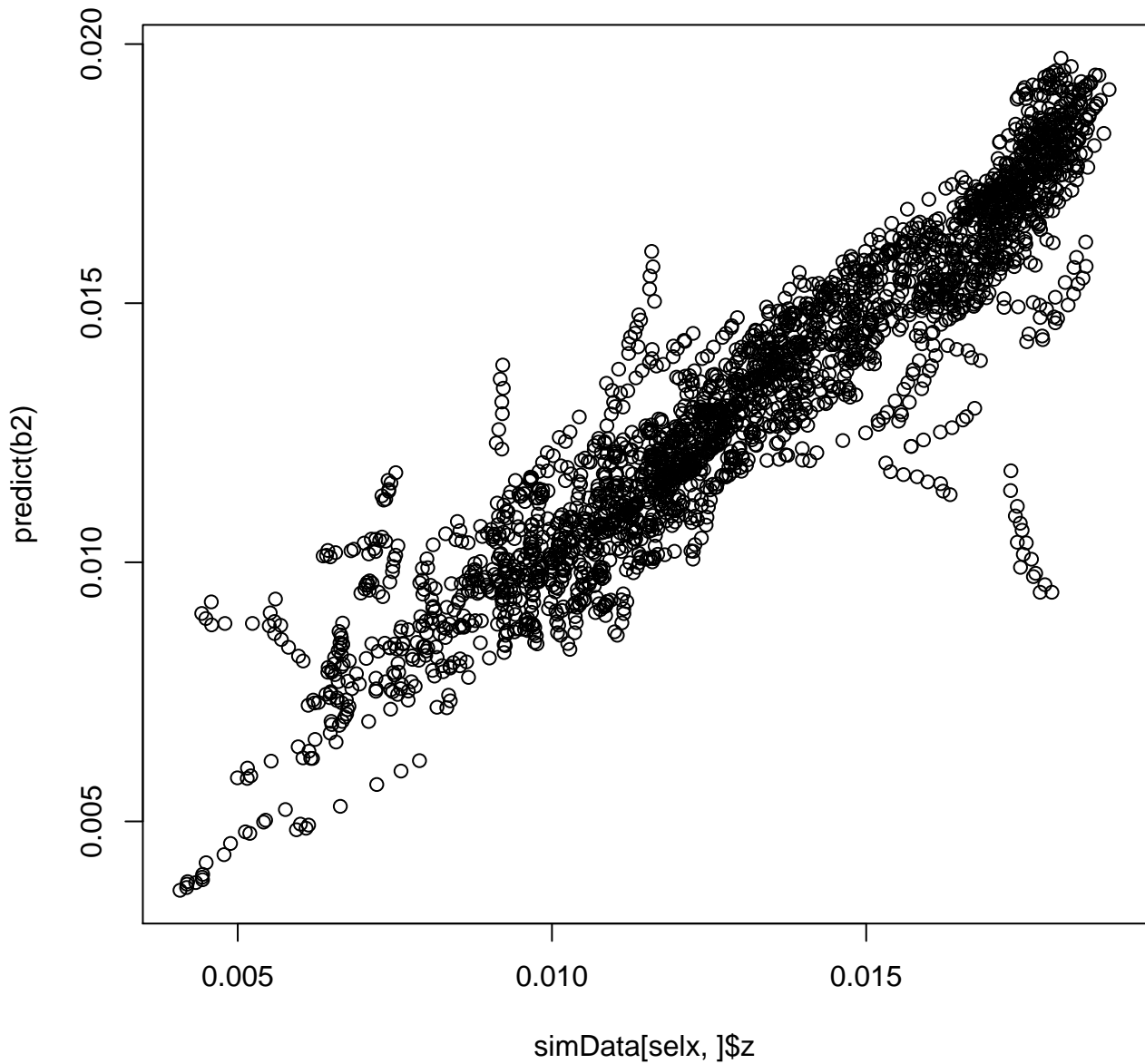

**Histogram of tree4d@data\$specH.mean.sim.gam**

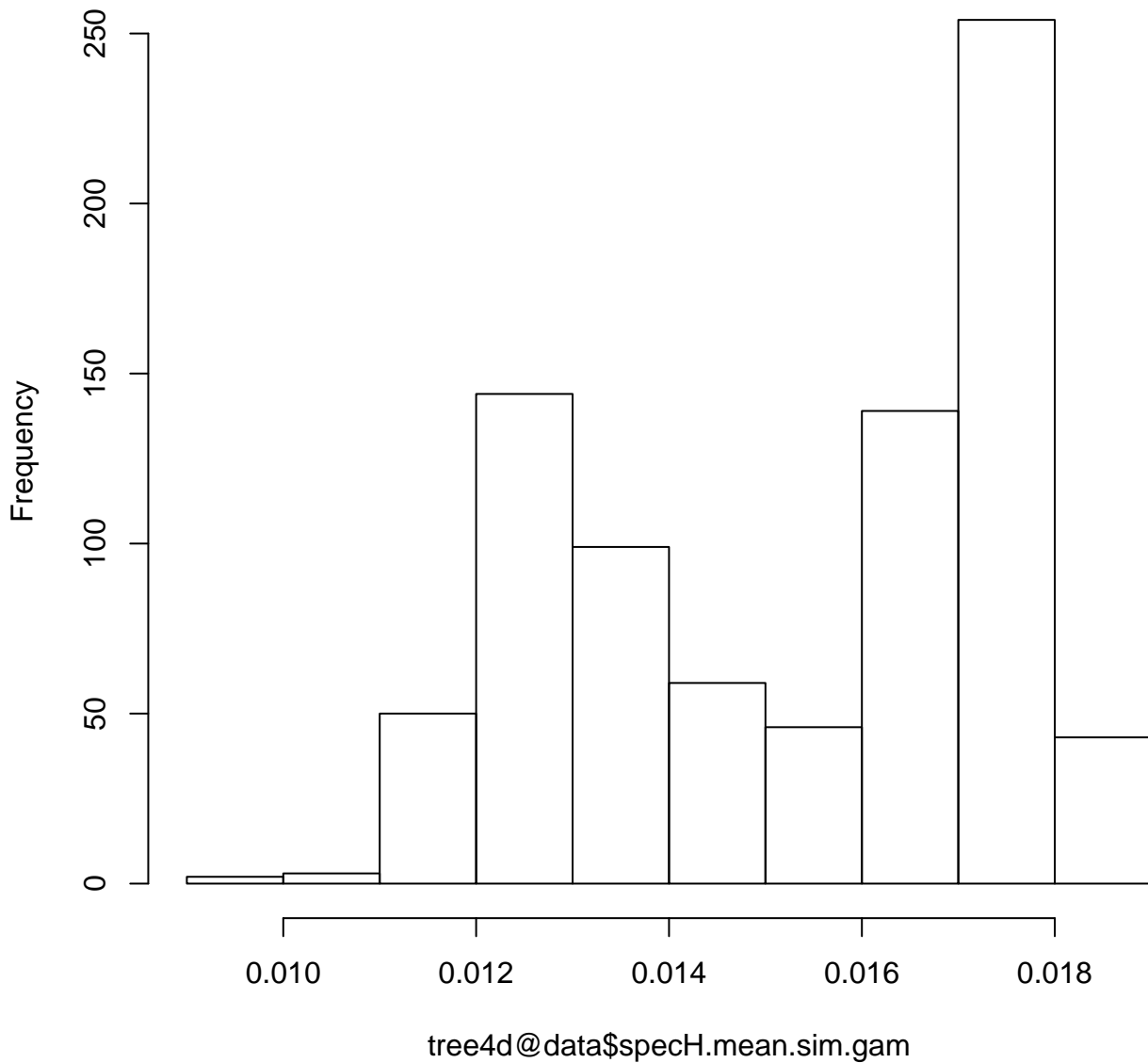

Supplement: Hartmann et al. supplementary material 4 — Hartmann et al. supplementary material [file S2513843X23000336sup004.zip › TonesClimateGeoPhylo_Public-1.0/processing/Rplots.pdf]

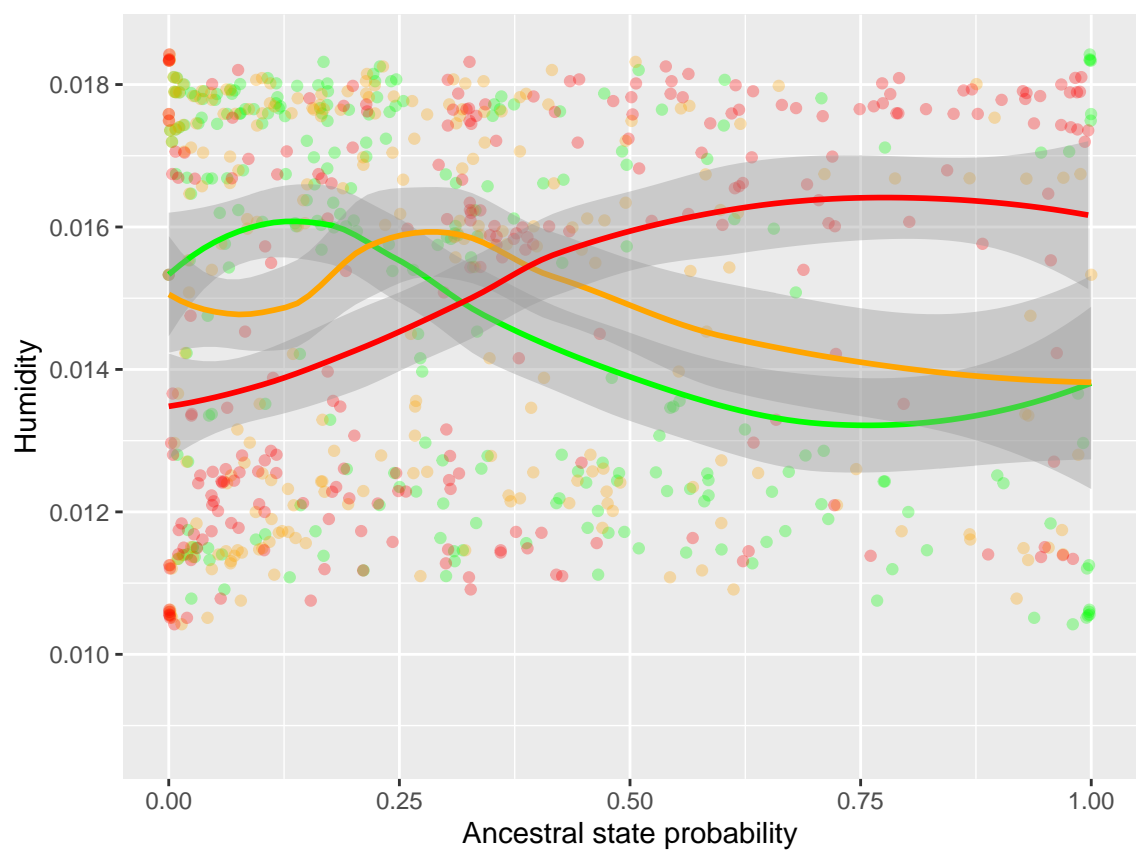

Supplement: Hartmann et al. supplementary material 4 — Hartmann et al. supplementary material [file S2513843X23000336sup004.zip › TonesClimateGeoPhylo_Public-1.0/results/BayesTraitsOutput/AncestralStateReconstruction/ANC_vs_Hum.pdf]

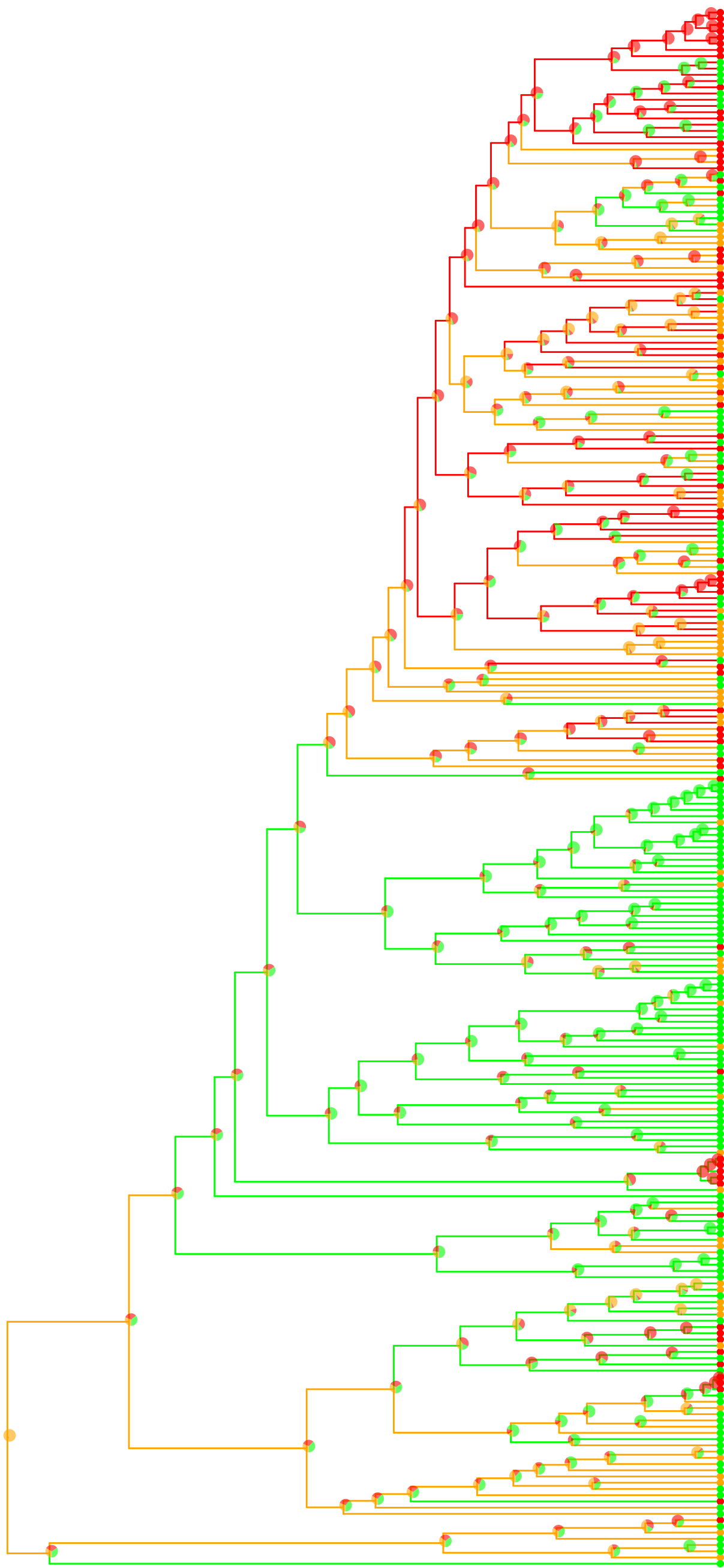

Supplement: Hartmann et al. supplementary material 4 — Hartmann et al. supplementary material [file S2513843X23000336sup004.zip › TonesClimateGeoPhylo_Public-1.0/results/BayesTraitsOutput/AncestralStateReconstruction/Phylogeny_Tones_Humidity_3cat_BayesTraits.pdf]

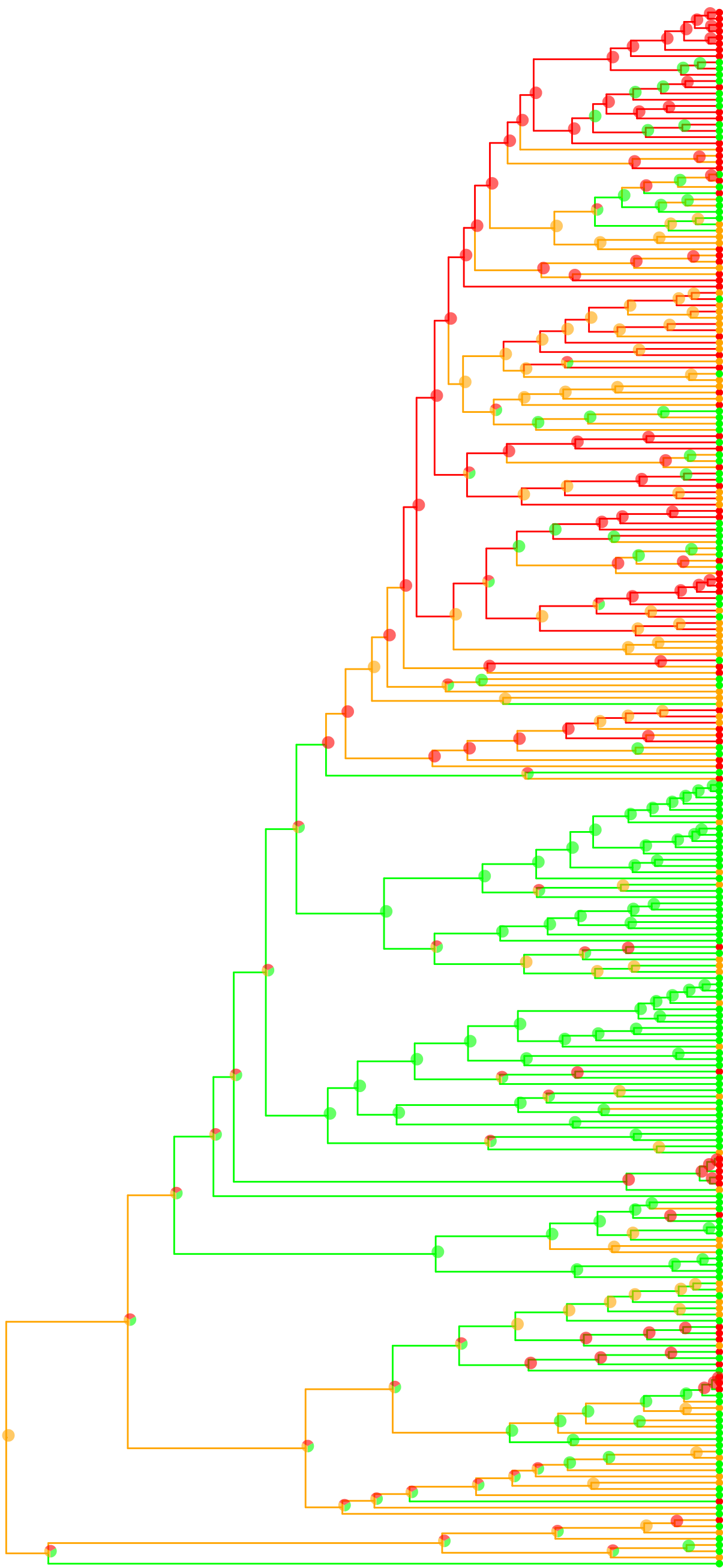

Supplement: Hartmann et al. supplementary material 4 — Hartmann et al. supplementary material [file S2513843X23000336sup004.zip › TonesClimateGeoPhylo_Public-1.0/results/BayesTraitsOutput/AncestralStateReconstruction/Phylogeny_Tones_Humidity_3cat_BayesTraits_MaxLikelihood.pdf]

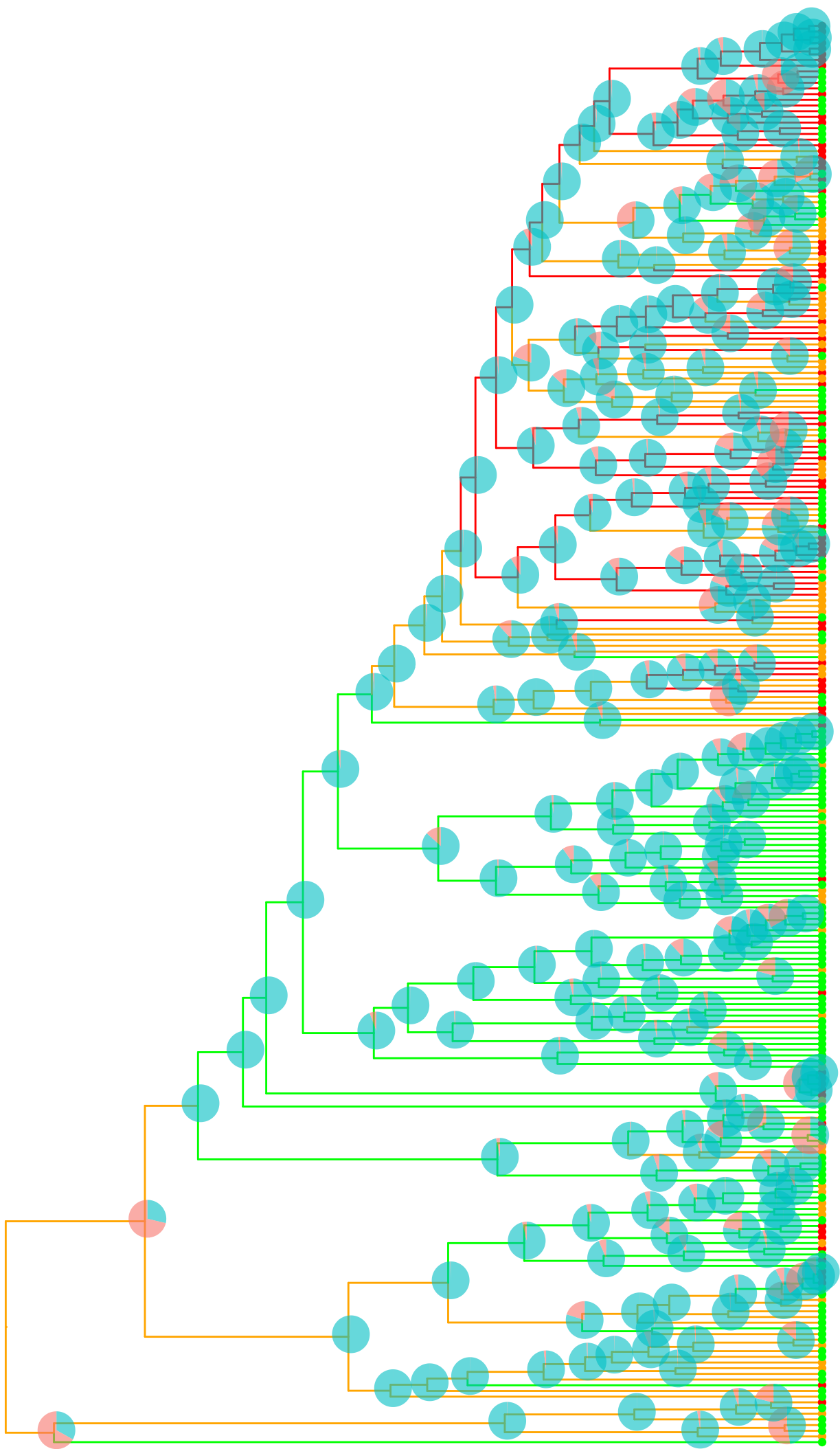

Supplement: Hartmann et al. supplementary material 4 — Hartmann et al. supplementary material [file S2513843X23000336sup004.zip › TonesClimateGeoPhylo_Public-1.0/results/BayesTraitsOutput/AncestralStateReconstruction/Phylogeny_Tones_Humidity_rate_BayesTraits.pdf]

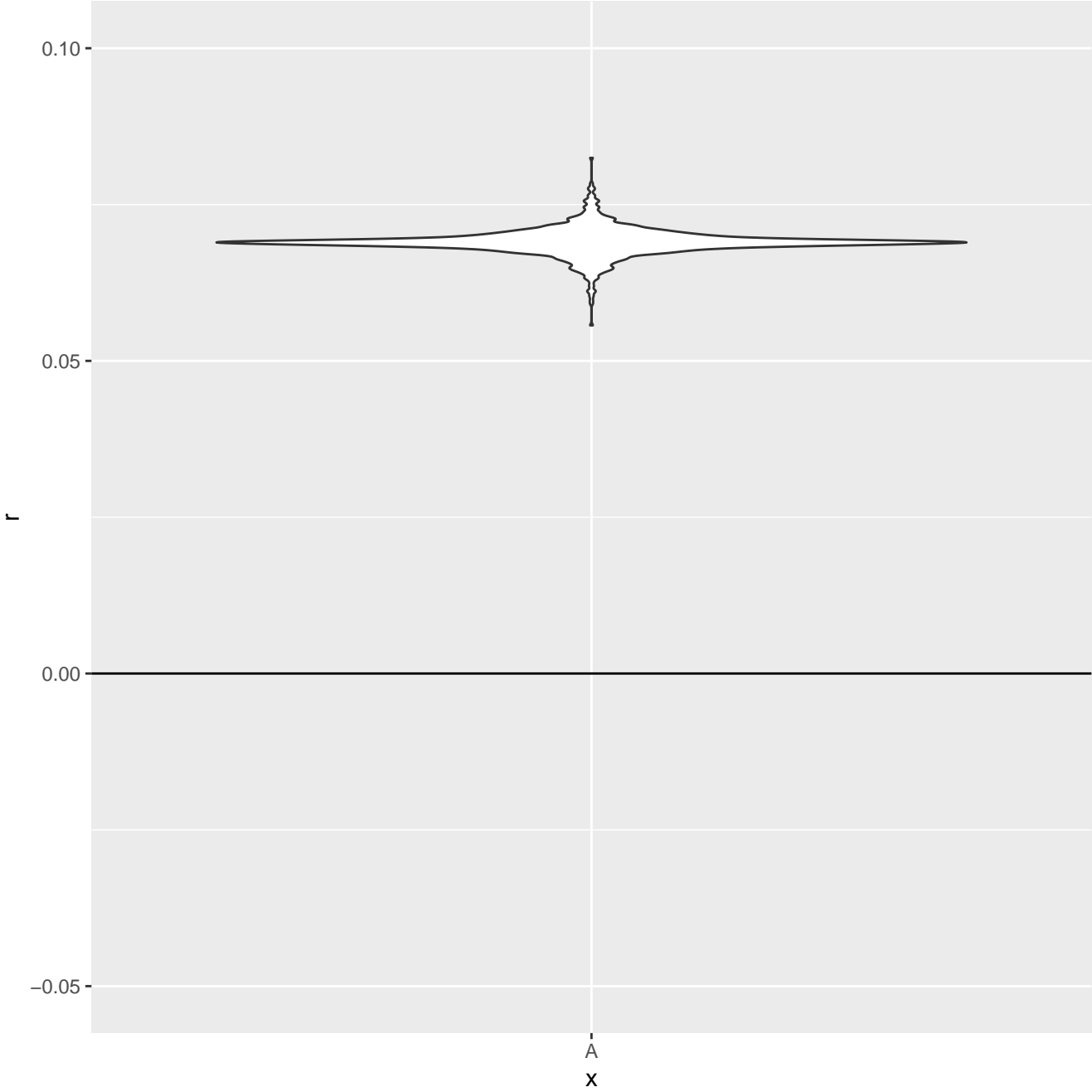

Supplement: Hartmann et al. supplementary material 4 — Hartmann et al. supplementary material [file S2513843X23000336sup004.zip › TonesClimateGeoPhylo_Public-1.0/results/BayesTraitsOutput/AncestralStateReconstruction/RawData.pdf]

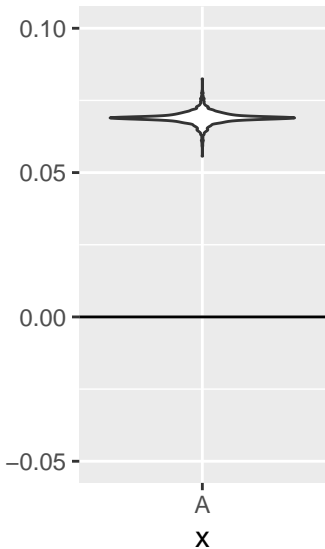

Supplement: Hartmann et al. supplementary material 4 — Hartmann et al. supplementary material [file S2513843X23000336sup004.zip › TonesClimateGeoPhylo_Public-1.0/results/BayesTraitsOutput/ContinuousA/Continuous_ModelA_R.pdf]

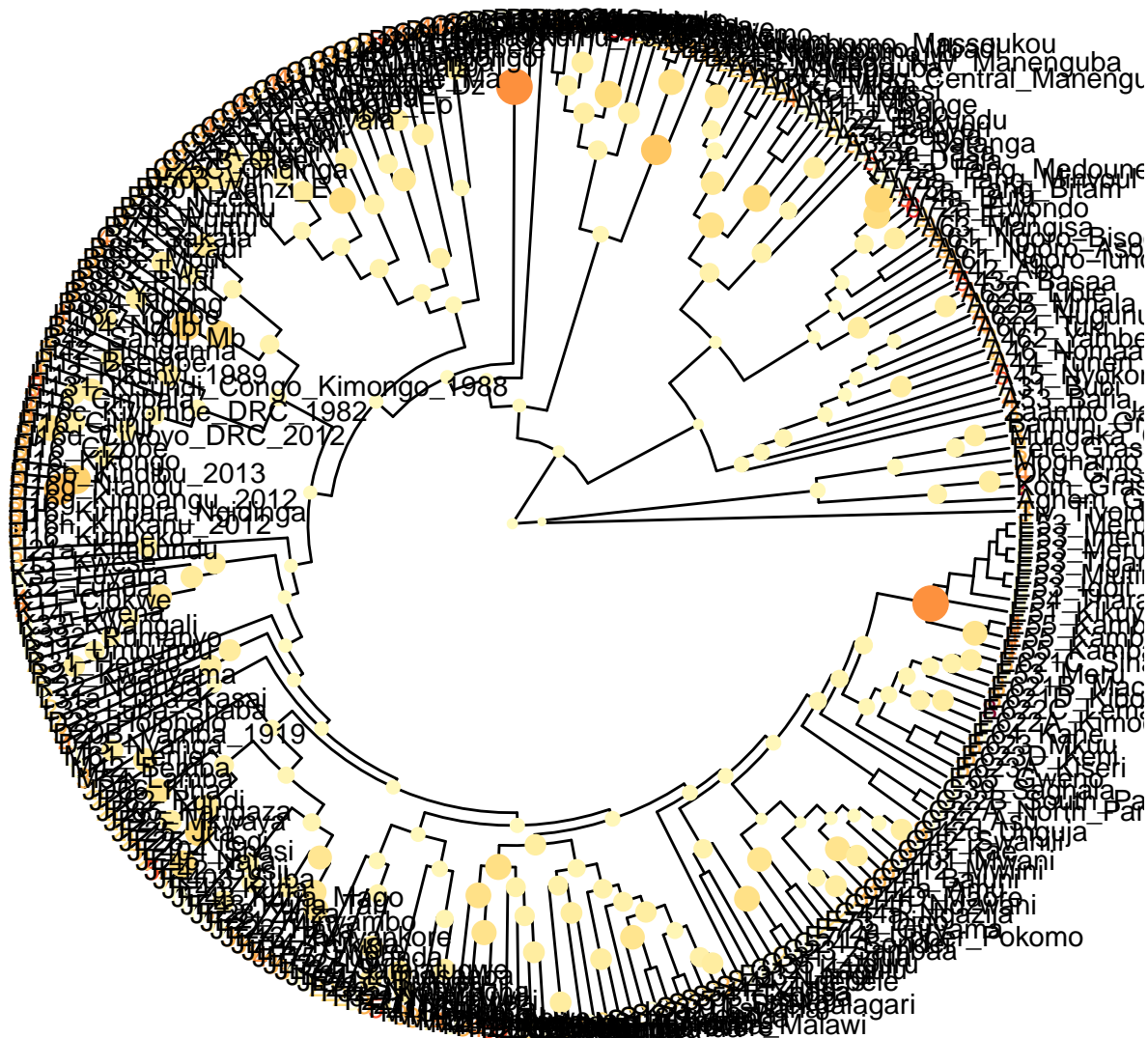

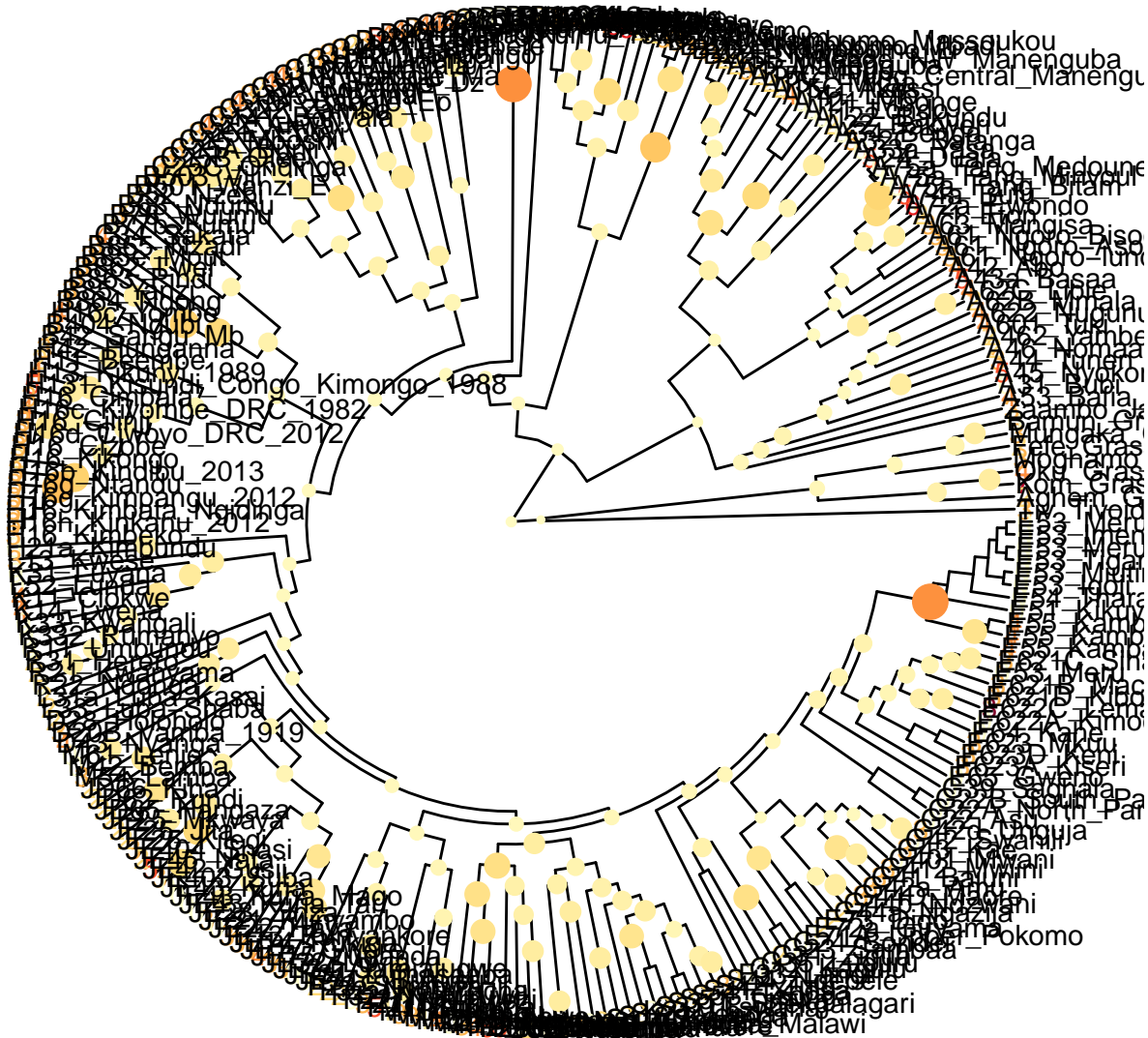

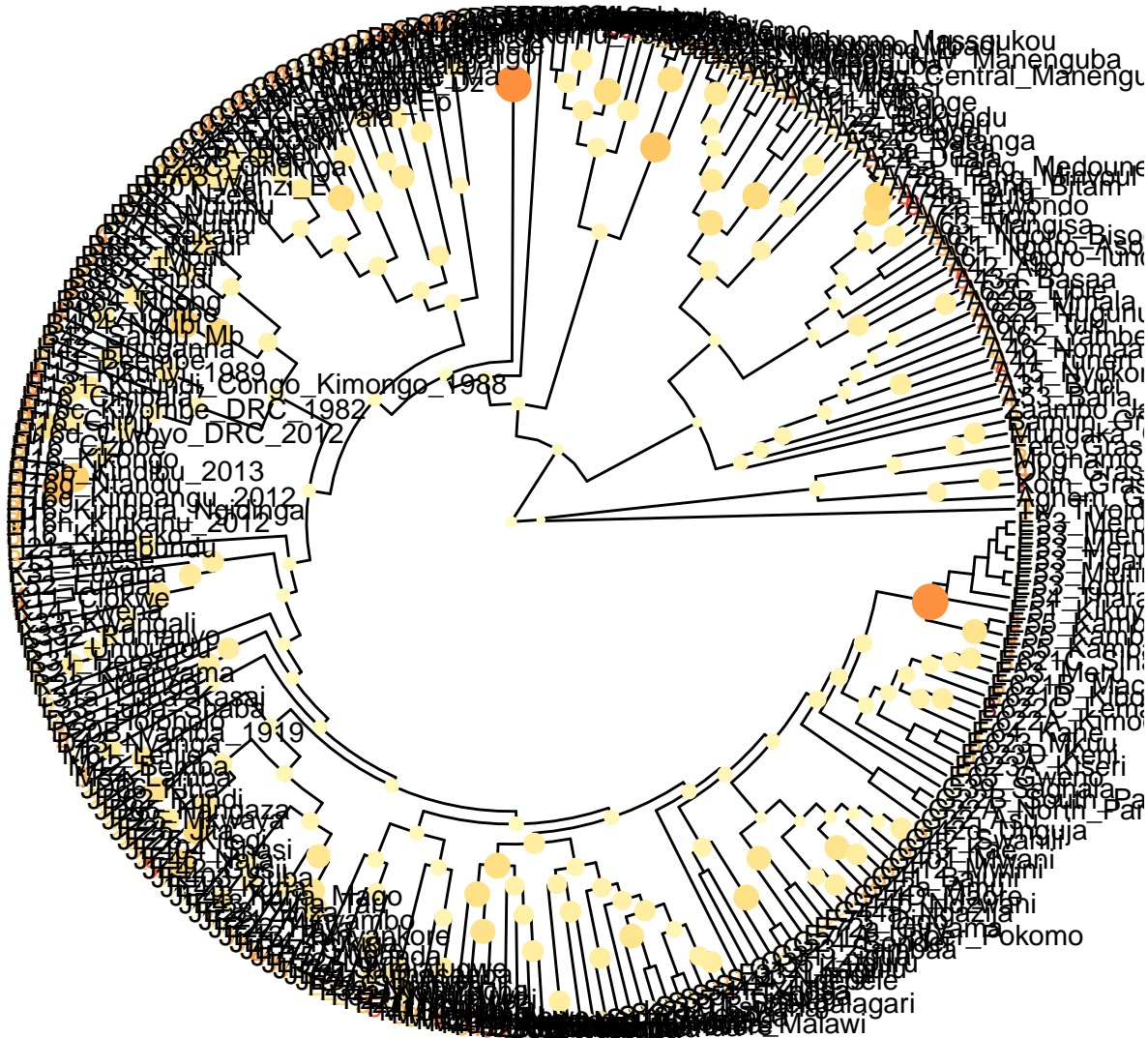

Supplement: Hartmann et al. supplementary material 4 — Hartmann et al. supplementary material [file S2513843X23000336sup004.zip › TonesClimateGeoPhylo_Public-1.0/results/BayesTraitsOutput/Discrete_Fossilised/DependentModel.pdf]

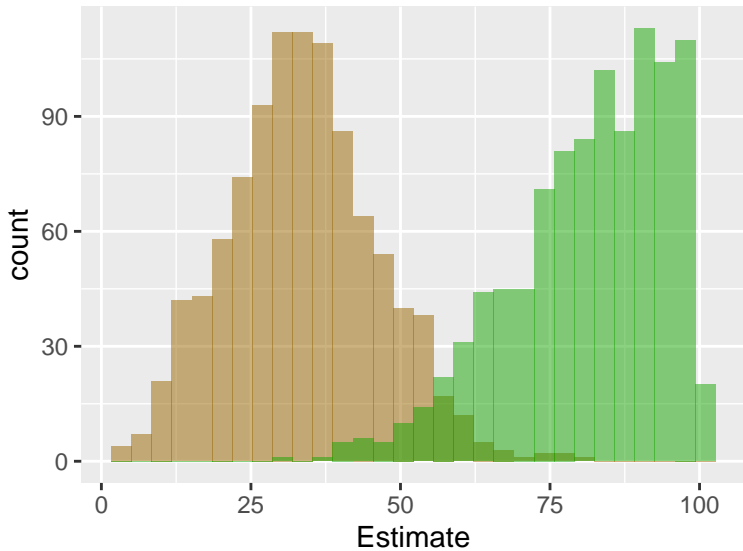

Supplement: Hartmann et al. supplementary material 4 — Hartmann et al. supplementary material [file S2513843X23000336sup004.zip › TonesClimateGeoPhylo_Public-1.0/results/BayesTraitsOutput/Discrete_Fossilised/GainTones.pdf]

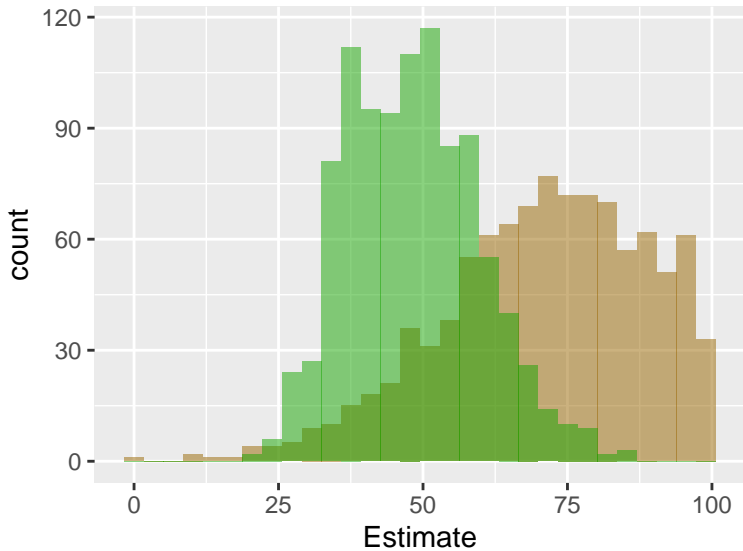

Supplement: Hartmann et al. supplementary material 4 — Hartmann et al. supplementary material [file S2513843X23000336sup004.zip › TonesClimateGeoPhylo_Public-1.0/results/BayesTraitsOutput/Discrete_Fossilised/LoseTones.pdf]
