# Supplementary material for: Investigating environmental effects on phonology using diachronic models
Source: Evol Hum Sci. 2024 Jan 3;6:e8. doi: 10.1017/ehs.2023.33 (PMC10955398; doi:10.1017/ehs.2023.33)
Supplement: Hartmann et al. supplementary material 4 — Hartmann et al. supplementary material [file S2513843X23000336sup004.zip › TonesClimateGeoPhylo_Public-1.0/results/graphs/PhylogenyData.pdf]

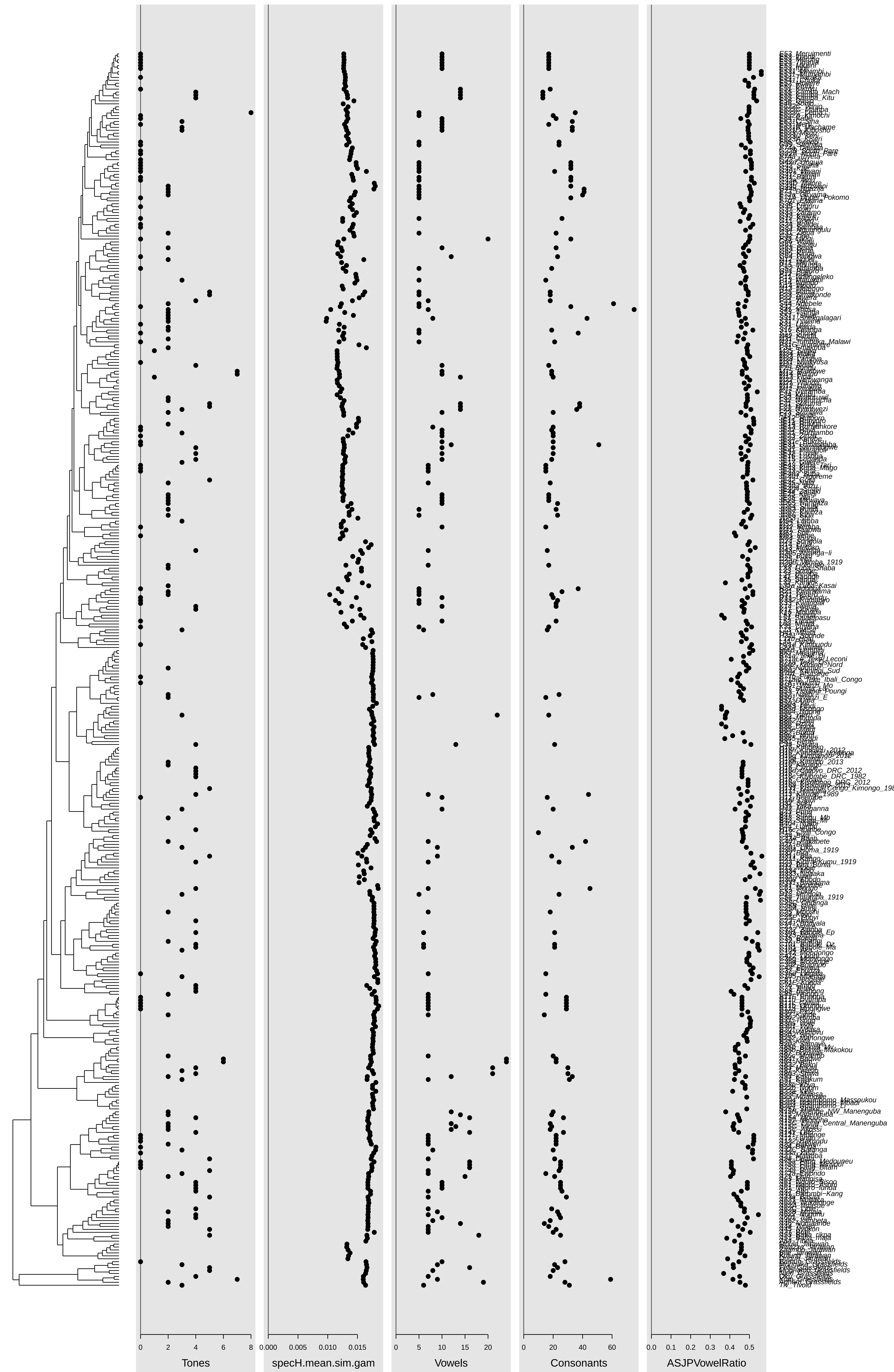

0 2 4 6 8
Tones
0.000 0.005 0.010 0.015
specH.mean.sim.gam
0 5 10 15 20
Vowels
0 20 40 60
Consonants
0.0 0.1 0.2 0.3 0.4 0.5
ASJPVowelRatio
