# Supplementary material for: Investigating environmental effects on phonology using diachronic models
Source: Evol Hum Sci. 2024 Jan 3;6:e8. doi: 10.1017/ehs.2023.33 (PMC10955398; doi:10.1017/ehs.2023.33)
Supplement: Hartmann et al. supplementary material 4 — Hartmann et al. supplementary material [file S2513843X23000336sup004.zip › TonesClimateGeoPhylo_Public-1.0/results/graphs/old/Phylogeny_Tones_Humidity.pdf]

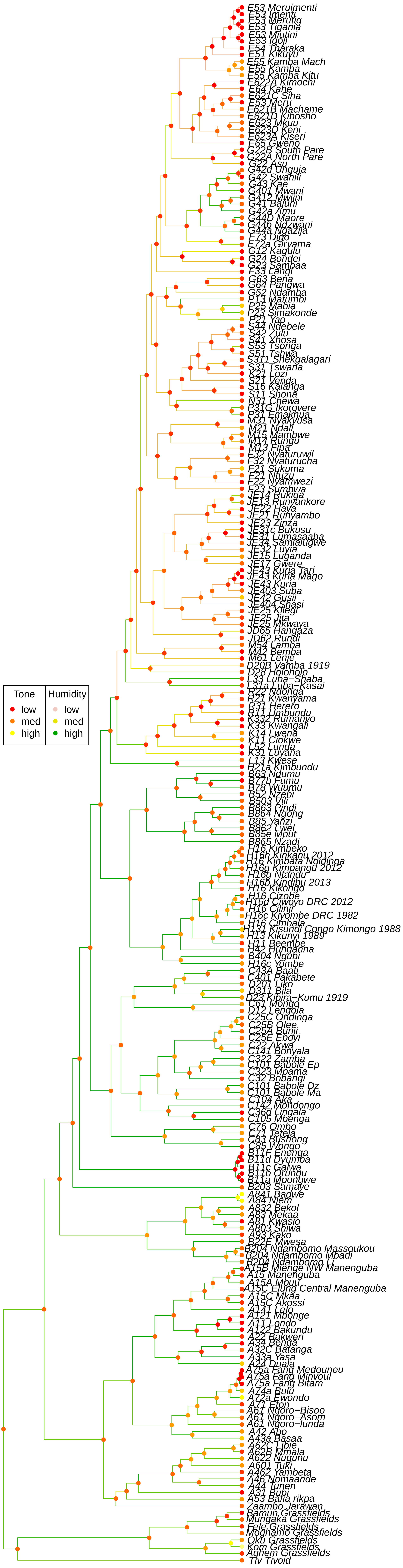
Tone
low
med
high
Humidity
low
med
high
E53 Meruimenti
E53 Imenti
E53 Merutig
E53 Tigania
E53 Miutini
E53 Igoji
E54 Tharaka
E51 Kikuyu
E55 Kamba Mach
E55 Kamba
E55 Kamba Kitu
E622A Kimochi
E64 Kahe
E621C Siha
E53 Meru
E621B Machame
E621D Kibosho
E623 Mkuu
E623D Keni
E623A Kiseri
E65 Gweno
G22B South Pare
G22A North Pare
G22 Asu
G42d Unguja
G42 Swahili
G43 Kae
G401 Mwani
G412 Mwiini
G41 Bajuni
G42a Amu
G44D Maore
G44b Ndzwani
G44a Ngazija
E73 Digo
E72a Giryama
G12 Kagulu
G24 Bondei
G23 Sambaa
F33 Langi
G63 Bena
G64 Pangwa
G52 Ndamba
P13 Matumbi
P25 Mabia
P23 Simakonde
P21 Yao
S44 Ndebele
S42 Zulu
S41 Xhosa
S53 Tsonga
S51 Tshwa
S311 Shekgalagari
S31 Tswana
K21 Lozi
S21 Venda
S16 Kalanga
S11 Shona
N31 Chewa
P31G Ikorovere
P31 Emakhua
M31 Nyakyusa
M21 Ndali
M15 Mambwe
M14 Rungu
M13 Fipa
F32 Nyaturuwil
F32 Nyaturucha
F21 Sukuma
F21 Ntuzu
F22 Nyamwezi
F23 Sumbwa
JE14 Rukiga
JE13 Runyankore
JE22 Haya
JE21 Runyambo
JE23 Zinza
JE31c Bukusu
JE31 Lumasaaba
JE34 Samialugwe
JE32 Luyia
JE15 Luganda
JE17 Gwere
JE43 Kuria Tari
JE43 Kuria Mago
JE43 Kuria
JE403 Suba
JE42 Gusii
JE404 Shasi
JE25 Kilegi
JE25 Jita
JE25 Mkwaya
JD65 Hangaza
JD62 Rundi
M54 Lamba
M42 Bemba
M61 Lenje
D20B Vamba 1919
D28 Holoholo
L33 Luba−Shaba
L31a Luba−Kasai
R22 Ndonga
R21 Kwanyama
R31 Herero
R11 Umbundu
K332 Rumanyo
K33 Kwangali
K14 Lwena
K11 Ciokwe
L52 Lunda
K31 Luyana
L13 Kwese
H21a Kimbundu
B63 Ndumu
B77b Fumu
B78 Wuumu
B52 Nzebi
B503 Vili
B863 Pindi
B864 Ngong
B85 Yanzi
B862 Lwel
B85e Mput
B865 Nzadi
H16 Kimbeko
H16h Kinkanu 2012
H16 Kimbata Ngidinga
H16g Kimpangu 2012
H16g Ntandu
H16b Kindibu 2013
H16 Kikongo
H16 Cizobe
H16d Ciwoyo DRC 2012
H16 Cilinji
H16c Kiyombe DRC 1982
H16 Cimbala
H131 Kisundi Congo Kimongo 1988
H13 Kikunyi 1989
H11 Beembe
H42 Hunganna
B404 Ngubi
H16c Yombe
C43A Baati
C401 Pakabete
D201 Liko
D311 Bila
D23 Kibira−Kumu 1919
C61 Mongo
D12 Lengola
C25C Ondinga
C25B Olee
C25A Bunji
C25E Eboyi
C22 Akwa
C141 Bonyala
C322 Zamba
C101 Babole Ep
C323 Mpama
C32 Bobangi
C101 Babole Dz
C101 Babole Ma
C104 Aka
C142 Mondongo
C36d Lingala
C105 Mbenga
C76 Ombo
C71 Tetela
C83 Bushong
C85 Wongo
B11F Enenga
B11d Dyumba
B11c Galwa
B11b Orungu
B11a Mpongwe
B203 Samaye
A841 Badwe
A84 Njem
A832 Bekol
A83 Mekaa
A81 Kwasio
A803 Shiwa
A93 Kako
B22E Mwesa
B204 Ndambomo Massoukou
B204 Ndambomo Mbadi
B204 Ndambomo Li
A15B Mienge NW Manenguba
A15 Manenguba
A15A Mbuu
A15C Elung Central Manenguba
A15C Mkaa
A15C Akossi
A141 Lefo
A121 Mbonge
A11 Londo
A122 Bakundu
A22 Bakweri
A34 Benga
A32C Batanga
A33a Yasa
A24 Duala
A75a Fang Medouneu
A75a Fang Minvoul
A75a Fang Bitam
A74a Bulu
A72a Ewondo
A71 Eton
A61 Ngoro−Bisoo
A61 Ngoro−Asom
A61 Ngoro−lunda
A42 Abo
A43a Basaa
A62C Libie
A62B Mmala
A622 Nugunu
A601 Tuki
A462 Yambeta
A46 Nomaande
A44 Tunen
A31 Bubi
A53 Bafia rikpa
Zaambo Jarawan
Bamun Grassfields
Mungaka Grassfields
Fefe Grassfields
Moghamo Grassfields
Oku Grassfields
Kom Grassfields
Aghem Grassfields
Tiv Tivoid
