# Supplementary material for: Investigating environmental effects on phonology using diachronic models
Source: Evol Hum Sci. 2024 Jan 3;6:e8. doi: 10.1017/ehs.2023.33 (PMC10955398; doi:10.1017/ehs.2023.33)

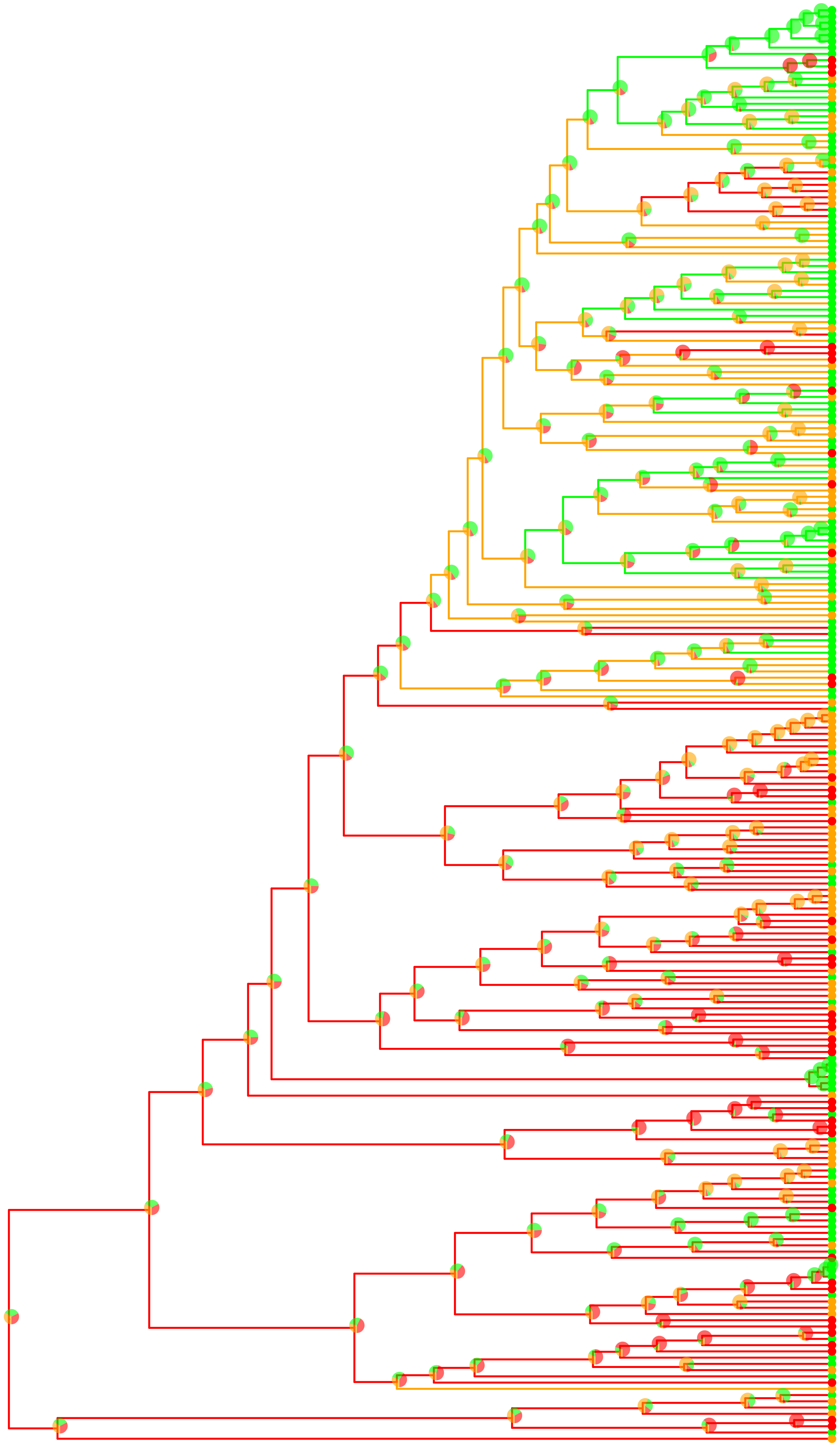

Supplement: Hartmann et al. supplementary material 4 — Hartmann et al. supplementary material [file S2513843X23000336sup004.zip › TonesClimateGeoPhylo_Public-1.0/results/graphs/old/Phylogeny_Tones_Humidity_3cat_BayesTraits copy.pdf]
